# Multidimensional Tracing of Error Sources and Structured Corrective Strategies for Leukocyte Count and Differential on Automated Hematology Analyzers

**DOI:** 10.3390/diagnostics16213578

**Published:** 2026-10-29

**Authors:** Ping Deng, Lianhui Yu, Haoting Zhan, Lin Zheng, Yipei Jing, Jiaying Fang, Min Zhang, Wei Wu

**Affiliations:** Department of Laboratory Medicine, Peking Union Medical College Hospital, Chinese Academy of Medical Sciences & Peking Union Medical College, Beijing 100730, China

**Keywords:** automated hematology analyzers, leukocyte count, spurious counts, misclassifications, error sources, corrective strategies, cross-platform analysis

## Abstract

Leukocyte count and differential are core indicators for clinical infection assessment, hematological disease diagnosis, and therapeutic monitoring. Currently, clinical testing heavily relies on automated hematology analyzers. Influenced by factors such as specimen characteristics, limited analyzer recognition capability, and improper manual operations, automated testing often produces unreliable results, interfering with clinical decision-making and threatening patient safety. Existing studies remain fragmented, lacking a multidimensional error-tracing framework and practical corrective strategies covering the entire testing workflow. A narrative review was conducted to summarize the causes, identification methods, and corrective strategies for unreliable leukocyte results. Relevant literature up to August 2026 was searched in PubMed/MEDLINE and CNKI. We categorized multidimensional error sources into specimen-related interferents, leukocyte morphological and developmental abnormalities, and preanalytical or systematic factors, and established a practical diagnostic workflow spanning from anomaly recognition to result reporting. By incorporating both legacy and current analytical systems, we compared the characteristic scattergram patterns of various interferents across analyzers with different detection principles, broadening the applicability to diverse clinical laboratories. To better ensure the reliability of corrective strategies and provide diverse alternative options, we not only summarized widely accepted routine practices but also included case-based experimental methods. The proposed multidimensional error-tracing system and practical diagnostic workflow significantly improve analytical accuracy and reduce clinical risks. However, current hematology analyzers cannot automatically identify or correct complex interferences. Future systems must integrate multi-platform advantages, optimize complex cell recognition capabilities, and enrich multidimensional scattergram information to overcome existing analytical limitations. Furthermore, integrating artificial intelligence technology holds immense potential to drive automated hematology analysis toward a more intelligent and precise direction.

## 1. Introduction

The leukocyte count and differential are among the most frequently requested tests in laboratory medicine, and they inform clinical decisions in some of the most time-critical scenarios—sepsis, chemotherapy monitoring, and hematological diseases. However, these results can be affected by a range of factors, including specimen characteristics, limited analyzer recognition capabilities, and improper manual operations. The resulting spurious counts and misclassification remain a daily challenge in laboratories worldwide.

Although numerous interferents have been reported, existing literature remains fragmented. Studies often isolate specific interferent features on single platforms, failing to clarify the intrinsic association between spurious count alterations and corresponding misclassification patterns. Moreover, laboratory professionals lack a practical diagnostic workflow to effectively trace these errors and implement evidence-based corrections.

**Figure 1:**
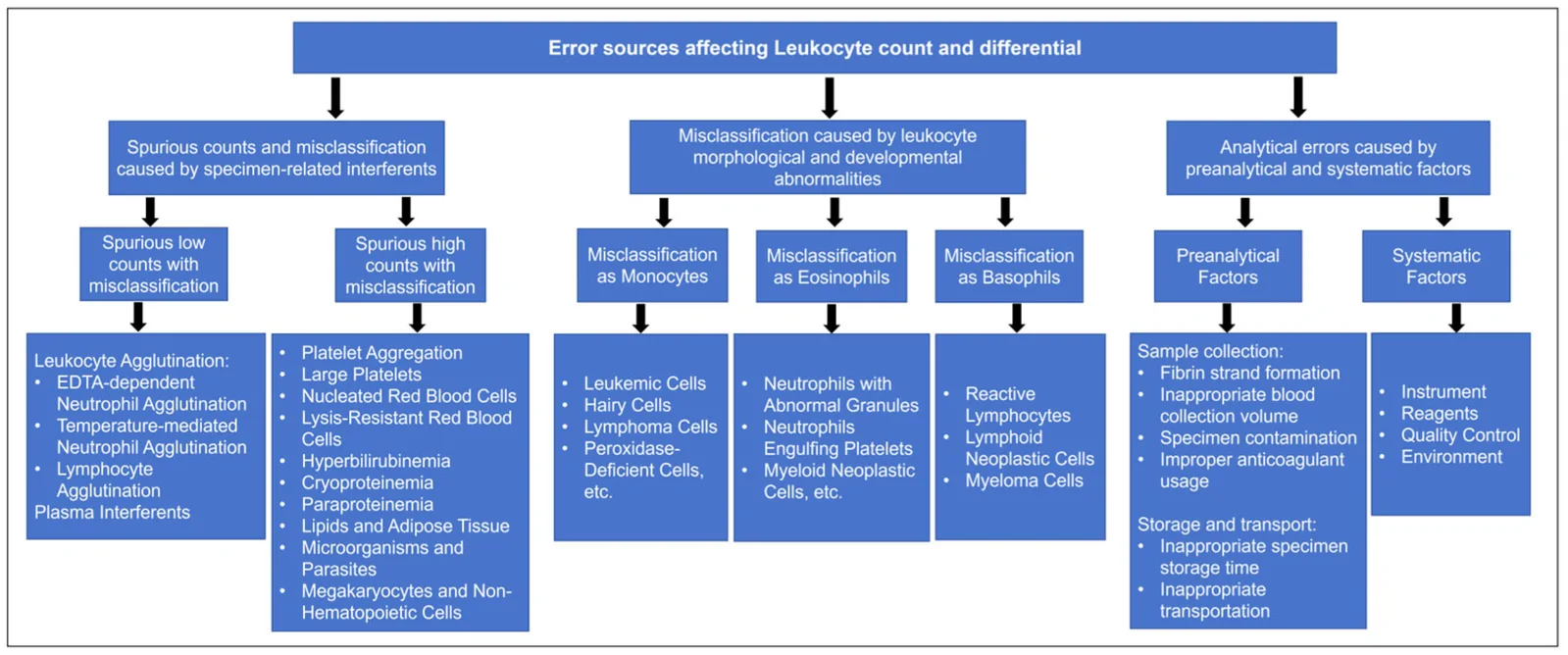
Overview of error sources contributing to unreliable leukocyte count and differential on automated hematology analyzers. The three main categories correspond to the organizational structure of this review. For leukocyte morphological and developmental abnormalities causing misclassification, only representative examples of morphologically abnormal cells are shown, with a comprehensive list of misclassification scenarios provided in Table 3 and the main text. The reasons for not separately subcategorizing neutrophil and lymphocyte misclassifications are explained in the main text. Other factors affecting leukocyte analysis that do not fall into the above three categories are discussed separately in Section 6.

To bridge these gaps, this review categorizes error sources into three main categories (Figure 1): specimen-related interferents causing spurious low and high leukocyte counts and misclassification, leukocyte morphological and developmental abnormalities causing misclassification, and preanalytical and systematic factors causing analytical errors.

**Figure 2:**
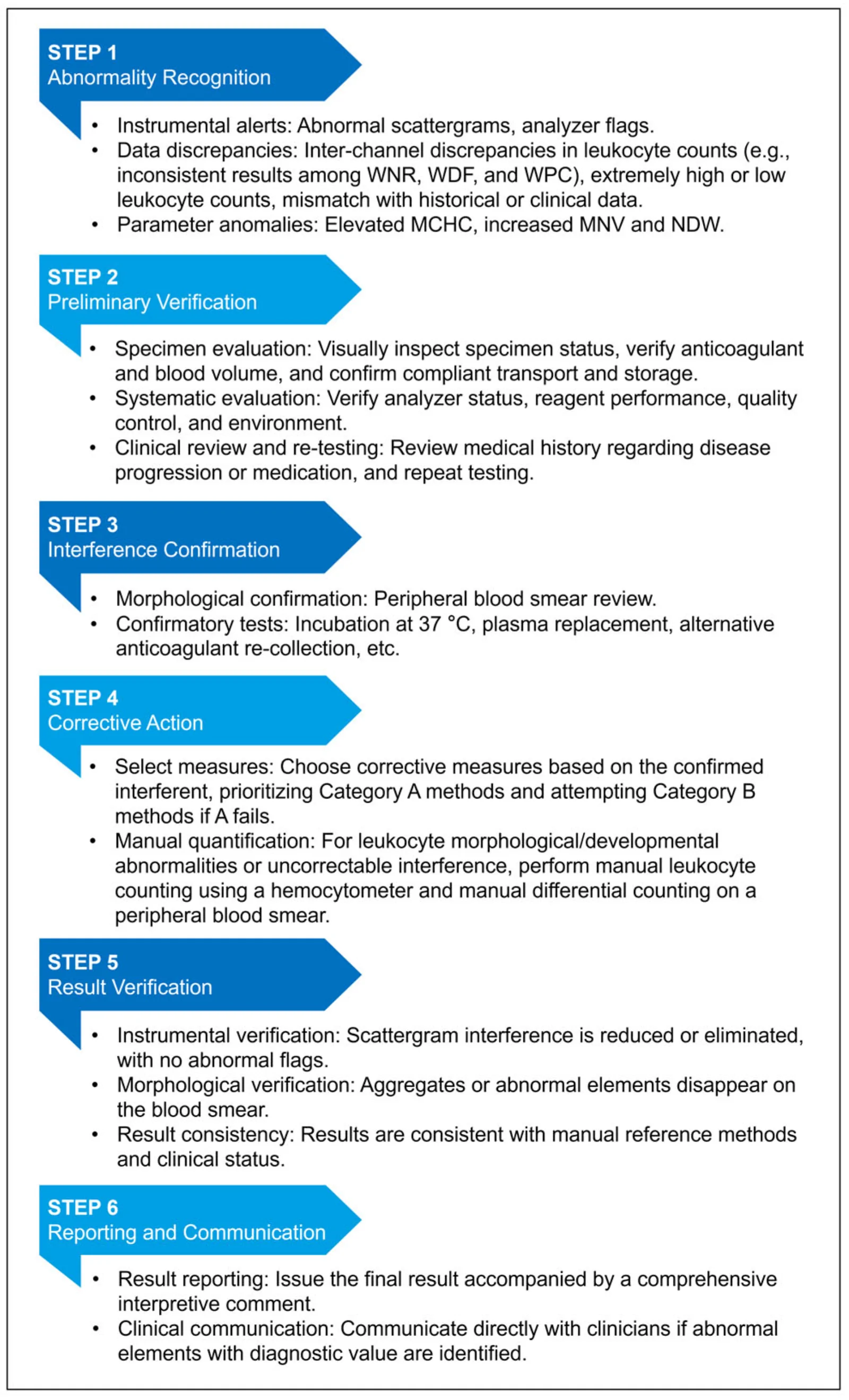
Practical diagnostic workflow for spurious leukocyte count and differential on automated hematology analyzers. Abbreviations: WNR, white cell nucleated red channel; WDF, white cell differential channel; WPC, white progenitor cell channel; MCHC, mean corpuscular hemoglobin concentration; MNV, mean neutrophil volume; NDW, neutrophil distribution width.

We further compare the scattergram features of the same interferent across platforms with distinct detection principles to broaden the applicability to diverse clinical laboratories. We also construct a practical diagnostic workflow spanning from anomaly recognition to result reporting (Figure 2) to provide actionable guidance for laboratory professionals in daily practice. This review aims to transform fragmented knowledge of interferents into a practical operational guide, promoting the transition of leukocyte analysis from empirical judgment to evidence-based correction.

## 2. Narrative Review Methodology

This article was designed as a narrative rather than a systematic review. The literature was identified through searches of PubMed/MEDLINE, complemented by manual screening of the reference lists of relevant reviews, guidelines, and consensus statements. In addition, CNKI was searched to enhance coverage of interfering factors and capture region-specific evidence. Literature published up to August 2026 was considered. Search terms included combinations of “leukocyte count,” “leukocyte differential count,” “spurious counts,” “pseudoleukocytosis,” “pseudoneutropenia,” “pseudolymphocytosis,” “pseudomonocytosis,” “pseudoeosinophilia,” “pseudobasophilia,” “interference,” “misclassification,” “correction,” “scattergram,” and “hematology analyzers.” For CNKI, corresponding Chinese terms were used (see Supplementary Table S1).

Studies were selected for their relevance to the interfering factors that cause unreliable leukocyte counts and differential results, the scattergram patterns these factors produce on different analyzers, and the corrective strategies for such interference. Priority was given to reviews; guidelines and consensus statements provided clinical context and evidence. Original research articles and case reports were also considered. Conference abstracts, editorials, and letters without peer review were excluded. Animal studies were excluded because the review addresses human clinical laboratory practice. A total of 107 articles were included. Four Chinese-language articles from CNKI were included for unique case data unavailable in English. As this is a narrative review, no formal systematic-review protocol, PRISMA-based selection, risk-of-bias assessment, or meta-analysis was performed. To help readers assess the present-day applicability of the described interference patterns and corrective strategies, the detection principles and relevant information of the analyzers discussed in this review are summarized in Supplementary Table S2.

## 3. Spurious Leukocyte Counts and Misclassification Caused by Specimen-Related Interferents

Leukocyte differential counting employs principles such as volume, conductivity, and scatter (VCS) technology, sheath-flow impedance combined with semiconductor laser flow cytometry, multi-angle polarized light scattering (MAPSS), laser scatter combined with peroxidase staining, and dual-sheath flow combined with cytochemical staining. These methods all rely on physical and chemical signals to achieve cell counting and classification. Specimen interferents (e.g., leukocyte aggregation, cryoglobulin, and platelet aggregation) can disrupt or mimic these signals, causing spurious low or high leukocyte counts with characteristic misclassification patterns [1,2,3]. The scattergram, the core visual interface, reveals shifts in normal cell clusters, population fusion, or abnormal clusters. Combined with instrument flags, these features provide key clues for anomaly detection. For cross-platform comparison, histogram/scattergram features and corrective measures for interferents on analyzers with different principles are summarized in Table 1 (Spurious low leukocyte counts combined with leukocyte misclassification) and Table 2 (Spurious high leukocyte counts combined with leukocyte misclassification).

### 3.1. Spurious Low Leukocyte Counts Combined with Misclassification

#### 3.1.1. Leukocyte Agglutination

Peripheral blood leukocyte agglutination is rare, with aggregates often too large to be counted, yielding spuriously low leukocyte counts. It involves neutrophils, lymphocytes, or other cell types. Occurrence is often EDTA- and/or low temperature-dependent, yet some cases lack an identifiable trigger [1,2,3]. It is suspected from fluctuating leukocyte counts or abnormal scattergram/histogram findings, and confirmed by blood smear microscopy. Although reported in various diseases, its disease associations and mechanisms remain unclear; triggers can differ even within the same disease [2]. Further investigation is therefore required.

##### EDTA-Dependent Neutrophil Agglutination

EDTA-K2, recommended by the International Council for Standardization in Haematology (ICSH) for its good anticoagulation and minimal morphological impact, can induce blood cell aggregation in practice. EDTA-dependent neutrophil agglutination is a rare in vitro phenomenon causing spuriously low leukocyte counts [4]. This must be distinguished from in vivo leukoembolization due to complement-activated granulocyte aggregation [36]. It has been reported in cirrhosis, infections, autoimmune diseases, uremia, immunosuppressive states, and various malignancies [1,9].

Some studies suggest that during EDTA-dependent neutrophil agglutination, analyzers employing differential lysis to generate bare nuclei yield relatively accurate results, whereas differential channel counts from other principles are mostly underestimated, with abnormal scattergrams and flags [4,5]. The fundamental interference mechanism is that leukocyte aggregation hinders accurate counting, thereby causing spuriously low leukocyte counts and misclassification [1]. It should be noted that large aggregates exceeding the leukocyte volume threshold set by the analyzer are classified as non-leukocyte particles and are not included in the count. Only smaller aggregates interfere with counting, leading to spuriously low results [5,6].

On impedance analyzers, a high-density zone above the polymorphonuclear neutrophil (PMN) area or difficult classification raises suspicion, typically generating “immature granulocyte” or “band cell” flags [4,5,6,7]. On analyzers employing laser scatter combined with peroxidase staining, a band of dots may appear in the upper right corner of the leukocyte differential scattergram. Because the aggregates contain large numbers of PMNs, they are reported as high-peroxidase cells/particles and trigger a high peroxidase content flag [5,6]. With VCS technology, the neutrophil cluster shifts toward and overlaps the monocyte region, attributed to reduced light scatter and/or increased volume, accompanied by markedly elevated mean neutrophil volume (MNV) and neutrophil distribution width (NDW) [8].

Upon these abnormal scattergrams or flags, immediate blood smear microscopy is advised. Smear edges show clumps of segmented neutrophils, within which immature granulocytes, lymphocytes, or monocytes may sometimes be observed [5,6,8]. Corrections include changing the anticoagulant, adding kanamycin, vortex-mixing, or immediately diluting fingerstick blood [1,4,5,9,10,11]. The degree of spurious reduction may correlate with the number and size of aggregates. Some cases may even be misdiagnosed as neutropenia or agranulocytosis, underscoring the necessity of accurate recognition for correct reporting.

##### Temperature-Mediated Neutrophil Agglutination

Temperature-mediated neutrophil agglutination is less frequently reported than EDTA-dependent agglutination. Although the triggers differ, the smaller aggregates formed by both can interfere with analyzer detection, causing spuriously low leukocyte counts and misclassification [12,13,14,37]. In vitro, blood smears from EDTA-anticoagulated samples at room temperature showed numerous neutrophil aggregates, and the leukocyte count was low. After 37 °C incubation, the count recovered, and the aggregates disappeared. The same pattern was observed with sodium citrate or heparin. Moreover, mixing healthy donor blood with patient serum or plasma at room temperature induced aggregation, suggesting the presence of leukoagglutinins [12,37]. Flow cytometry detected IgM on patient neutrophils, and some investigators believe IgM antibodies contribute to agglutination [13,14].

This agglutination can also interfere with analyzers of different principles, producing flags and scattergram abnormalities similar to those described in the EDTA-dependent Neutrophil Agglutination Section. Additionally, it may trigger a T2 flag on the white blood cell (WBC) histogram of the Sysmex KX-21 [12]. On the Coulter S-Plus IV, it manifests as abnormal granulocyte and monocyte distribution [13]. Markedly elevated VCS parameters (MNV, NDW) provide auxiliary clues [12].

When these abnormal scattergrams or flags appear, a blood smear must be prepared immediately for microscopic confirmation. Effective corrective measures include incubating the sample at 37 °C for 10–20 min, anti-IgM or dithiothreitol (DTT) treatment of plasma, plasma replacement (an in vitro sample preprocessing technique that replaces the patient’s plasma with saline, healthy donor plasma, or other matrices before reanalysis), and prompt testing of capillary blood [1,6,12,14].

##### Lymphocyte Agglutination

Lymphocyte agglutination is a rare, usually EDTA-dependent phenomenon, occurring far less frequently than neutrophil agglutination. It is one of the important interfering factors causing spuriously low leukocyte counts on impedance analyzers. It has been reported in chronic lymphocytic leukemia (CLL) [15], splenic lymphoma with villous lymphocytes [6], non-Hodgkin lymphoma [38], chronic myelomonocytic leukemia [39], and persistent polyclonal B-lymphocytosis [16]. Some researchers believe that arachidonic acid, leukotriene B4, and epinephrine may be involved in lymphocyte agglutination [5,6,15,40,41]. Compared with individual lymphocytes, agglutinated cells exhibit increased volume and complexity. Large aggregates evade detection, causing spuriously low counts. Smaller aggregates interfere with classification and trigger abnormal histogram flags [5,41]. On the Technicon H6000, which classifies leukocytes based on peroxidase staining and cell volume, lymphocyte agglutination appears as an abnormal dot band above the monocyte–basophil area on the peroxidase (Perox) scattergram [15]. Lymphocyte agglutinates seen on blood smears may be composed of normal lymphocytes, abnormal lymphocytes, or both [38]. An example in a CLL patient is shown in Figure S1. Immediate dilution of fingerstick blood or switching to sodium citrate or heparin usually effectively reduces this phenomenon [5,6,16].

In a small subset of patients, aggregates of neutrophils, lymphocytes, and monocytes coexist. These are difficult to identify by scattergrams and flags and must be detected by smear microscopy. Regardless of trigger or cell type, leukocyte agglutination requires smear confirmation. After corrective measures, a repeat smear must verify the disappearance of aggregates before results are considered reliable [1].

#### 3.1.2. Plasma Interferents

Plasma interferent-induced spurious leukopenia is extremely rare and clinically difficult to recognize. Most reported cases have been associated with Sysmex analyzers, which employ sheath-flow impedance combined with semiconductor laser flow cytometry. These cases were discovered due to significant discrepancies between the white cell differential (WDF; also referred to as DIFF) and white cell nucleated (WNR)/basophil (BASO) channel leukocyte counts [17,18,19,42,43,44]. A common feature is that the WNR scattergram shows numerous dots near the *X*-axis or reduced dots in the leukocyte counting area, with the count much lower than the smear estimate. The WDF channel shows no interfering dots, and its count matches the smear. Qian et al. [17] demonstrated that plasma replacement markedly reduced the abnormal dots in the WNR scattergram and normalized the count, while the WDF channel remained unchanged. They also observed that mixing patient plasma with WNR reagent immediately produced an insoluble white clot, indicating that certain plasma components form clots in the strongly acidic lysing reagent and adhere to leukocytes, causing spurious low counts. Tantanate [18] retested the same sample with a Beckman Coulter LH780 and obtained smear-consistent results without flags, suggesting VCS technology may be unaffected by this interference. For paraneoplastic syndrome, Sysmex hypothesized that hyaluronic acid or hyaluronidase oversecreted by these patients may polymerize in the acidic WNR reagent to form mucin clots, leading to leukocyte aggregation or excessive breakdown. This remains unverified in vitro [44]. Therefore, when a large discrepancy between WNR and WDF channel leukocyte counts is observed on Sysmex analyzers, smear microscopy should be performed for clarification. If the smear estimate matches the WDF result, the WDF result may be accepted [18]. If necessary, alternative analyzers or predilution mode can be used to reduce interference [18,19].

### 3.2. Spurious High Leukocyte Counts Combined with Misclassification

#### 3.2.1. Platelet Aggregation and Large Platelets

Platelet aggregation arises from difficult venipuncture, inappropriate anticoagulation, or EDTA-dependent pseudothrombocytopenia (EDTA-PTP). Traditional impedance analyzers gate leukocytes at 35–450 fL. Platelet aggregates exceeding 35 fL are miscounted as leukocytes and often misclassified as lymphocytes, yielding spuriously low platelet counts and spuriously high leukocyte counts [1,45]. The WBC histogram shows an elevated start at 35 fL [20]. The platelet histogram displays an irregular peak, an upturned tail, or an abnormal contour. Analyzers employing multi-channel, multi-method approaches are equipped with dedicated algorithms for identifying platelet aggregation or independent channels that specifically recognize platelets, providing alerts to laboratory personnel. On Sysmex analyzers, platelet aggregation produces an abnormal Platelet-Impedance (PLT-I) histogram (Figure S2A) and characteristic abnormal signals on the Platelet-Fluorescence (PLT-F) scattergram (Figure S2B), triggering “platelet aggregation” and “abnormal platelet histogram” flags. Some platelet aggregation specimens can yield discordant leukocyte counts across the WDF, WNR, and white progenitor cell (WPC) channels. On the Siemens ADVIA 2120, platelet aggregation may cause a large discrepancy between the leukocyte counts from the basophil/lobularity (BASO) channel and the Perox channel and form a characteristic abnormal scatter cluster on the Perox scattergram (Figure S3), directly interfering with leukocyte differential [5]. Corrections include changing anticoagulant, vortex mixing, and incubation [1,21].

Some large platelets have volumes comparable to those of leukocytes and are mistakenly counted as leukocytes by traditional impedance analyzers, producing spuriously low platelet counts and spuriously high leukocyte counts [1,6,20]. On Sysmex analyzers, such specimens show discordant leukocyte counts across the WDF, WNR, and WPC channels, accompanied by abnormal platelet histogram morphology and abnormal PLT-F scattergram distribution (Figure S4A,B). Characteristic interfering dots appear on the WDF and WNR scattergrams (Figure S4C,D). The analyzer simultaneously triggers “abnormal WBC scattergram” and “abnormal platelet histogram” flags. For specimens severely interfered with by large platelets, manual counting using a hemocytometer can be performed to obtain an accurate leukocyte count [1,5]. Histograms, scattergrams, and flags only suggest possible platelet aggregation or large platelets. Blood smear confirmation remains mandatory for final verification and for validating corrective measures.

#### 3.2.2. Nucleated Red Blood Cells (NRBCs)

A small number of NRBCs are seen in neonatal blood physiologically. Pathological conditions, including hemolytic anemia, severe thalassemia, and myelodysplastic syndromes (MDSs), can release large numbers into the peripheral blood. Traditional impedance analyzers lyse mature red blood cells for leukocyte counting. Unable to dissolve the NRBC nuclear membrane, these bare nuclei are erroneously counted as leukocytes. Their volume resembles that of lymphocytes, so they are frequently misclassified as such, producing a double peak in the lymphocyte region of the leukocyte histogram [22]. Analyzers employing laser scatter and peroxidase staining are also affected. NRBCs lack peroxidase activity and appear in the lower left of the Perox scattergram. When abundant, this region shows characteristic changes and interferes with leukocyte count and differential (Figure S5) [6]. Sysmex analyzers use the WNR channel’s fluorescent dye to differentially stain leukocyte and NRBC nucleic acids, enabling their separation. Nevertheless, specimens containing large numbers of NRBCs can still interfere with leukocyte count and differential, producing an abnormal distribution pattern on the WDF scattergram (Figure S6), mainly impairing lymphocyte classification. When such histogram or scattergram features accompany relevant flags, NRBCs should be strongly suspected, and a blood smear is required to confirm and assess the interference. If the NRBC count exceeds the analyzer’s automatic correction limit, manual counting is required, and the leukocyte count is corrected with the formula: corrected WBC = X × 100/(100 + Y), where X is the uncorrected count, and Y the NRBCs per 100 leukocytes in the differential [1].

#### 3.2.3. Lysis-Resistant Red Blood Cells (Lysis-Resistant RBCs) and Hyperbilirubinemia

Normal erythrocyte membranes consist of skeleton proteins and lipids (mainly phospholipid [PL] and cholesterol [CH]), with a CH/PL ratio of 0.8–1.0. In lytic reagents, normal erythrocytes are completely lysed into ghost cells that do not interfere with leukocyte detection. In pathological conditions such as hemoglobinopathies, liver disease, uremia, and post-chemotherapy states, membrane changes produce lysis-resistant RBCs that are counted as leukocytes, leading to spuriously high counts. Membrane lipid analysis of these lysis-resistant RBCs shows markedly reduced PL content, elevated CH content, and an increased CH/PL ratio, indicating altered membrane lipid structure [46]. This abnormality impairs the lytic reagent’s cleavage of membrane phospholipids and their separation from membrane proteins, rendering erythrocytes lysis-resistant. Lysis-resistant RBCs are frequently misclassified as lymphocytes [6,23]. On the Beckman Coulter DxH 900, the VCS scattergram shows a diffuse lymphocyte population with blurred boundaries, and an abnormal cluster of discrete dots appears in the lower left of this area, partially overlapping the lymphocytes, which can cause spuriously high lymphocyte counts [23,47]. On Sysmex analyzers, lysis-resistant RBCs cause a discrepancy in leukocyte counts between the DIFF and BASO channels, with the BASO channel showing a greater deviation [48]. The WNR channel scattergram may also show characteristic changes in some specimens (Figure S7). When such abnormalities are detected, high suspicion of lysis-resistant RBC interference should be raised, especially in post-chemotherapy cancer patients, since the spurious lymphocytosis may mask actual leukopenia. Diluting and retesting is a common corrective strategy [1,23]. Increasing the lytic reagent volume, prolonging lysis time, or using reagents with stronger lytic capacity may help reduce the interference, but specific conditions require experimental validation [6,24].

Hyperbilirubinemia can spuriously elevate hemoglobin (HGB), mean corpuscular hemoglobin (MCH), and mean corpuscular hemoglobin concentration (MCHC) via colorimetric interference [1]. Some hyperbilirubinemic patients also develop spuriously high leukocyte counts and misclassification. The mechanism involves incomplete lysis and abnormal light scatter. Incomplete lysis occurs because high bilirubin concentrations form stable free radicals, which undergo lipid peroxidation with unsaturated fatty acids in the erythrocyte membrane, leading to a reduction in membrane unsaturated fatty acids. This renders the membrane resistant to lysis by lytic reagents, and the unlysed erythrocytes are miscounted as leukocytes. The degree of interference intensifies with increasing bilirubin concentration [49]. Abnormal light scatter manifests as bilirubin-induced irregular changes in scattered light signals, preventing the analyzer from correctly identifying leukocyte populations and resulting in non-classification or misclassification [23]. On VCS analyzers, the scatter dots of different leukocyte populations overlap and lose their boundaries, directly causing differential failure or misclassification [23,25]. On the CELL-DYN 1800 analyzer (Coulter principle), some hyperbilirubinemic patients show spuriously high leukocyte counts accompanied by elevated absolute lymphocyte counts [50]. Thus, elevated bilirubin does not universally cause spuriously high leukocyte counts, and the affected leukocyte populations are non-specific. Sample dilution may reduce this interference in some cases [1,23].

#### 3.2.4. Cryoproteinemia and Paraproteinemia

**Figure 3:**
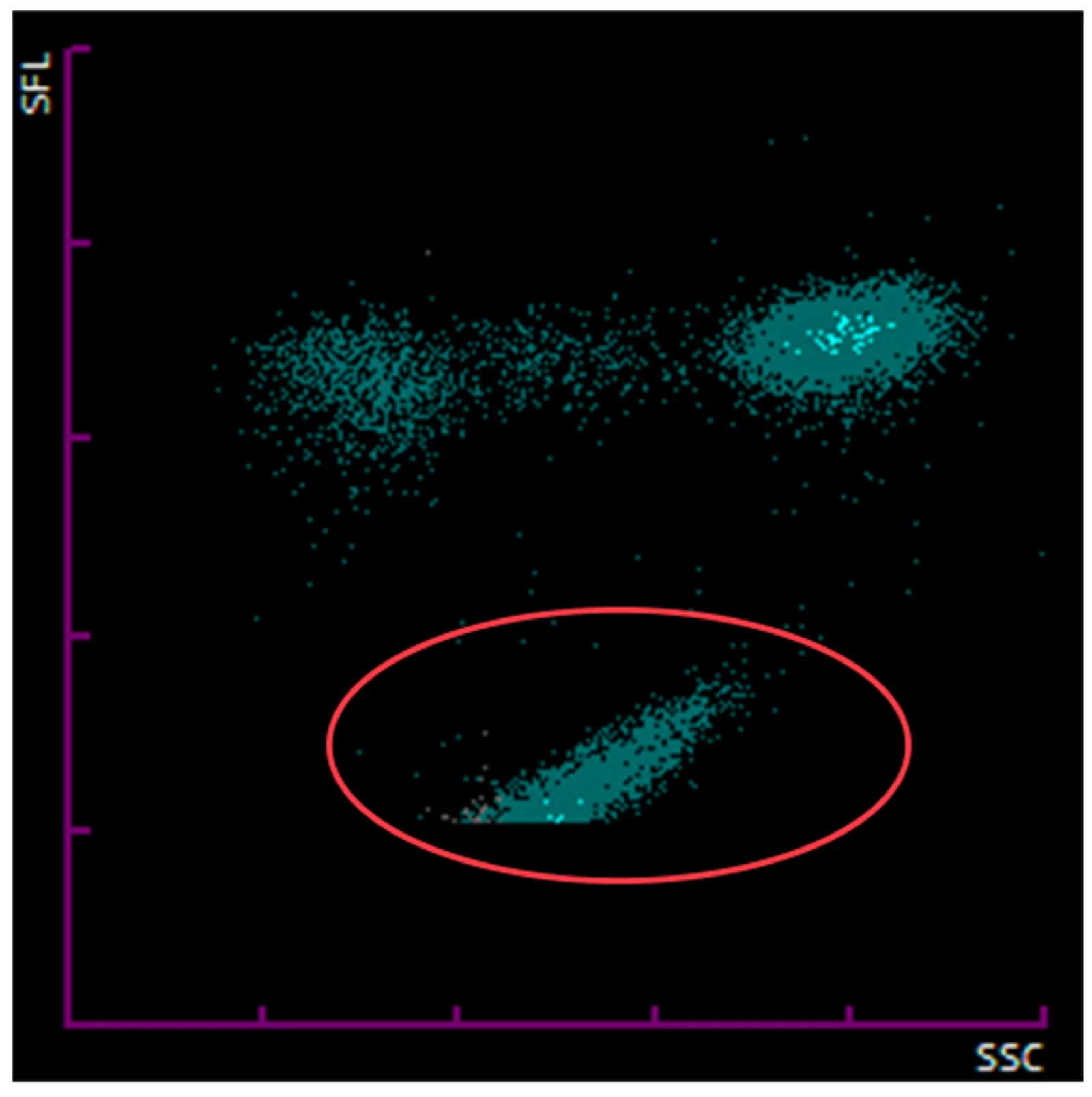
Cryoglobulin interference on the WPC scattergram of the Sysmex XN-9000. A distinct triangle-shaped abnormal dot cluster (circled in red) appears below the leukocyte population, indicative of cryoglobulin interference with the leukocyte count.

Cryoglobulins and cryofibrinogen are plasma proteins that precipitate at low temperatures and redissolve upon warming. Cryoglobulinemia is associated with immunoproliferative disorders, autoimmune diseases, and infectious diseases, especially hepatitis C. They can significantly interfere with complete blood counts, often causing spuriously high leukocyte and platelet counts and affecting red blood cell indices [1,6,51,52]. The core mechanism is that cryoglobulin particles aggregate to reach the leukocyte size range and are miscounted as leukocytes. On VCS analyzers, an abnormal small peak with an early take-off appears at the far left of the leukocyte volume histogram, indicating small particles counted within the leukocyte population [26]. On analyzers employing laser scatter combined with peroxidase staining, abnormal interfering dots appear on the Perox scattergram, located above the lymphocyte or neutrophil area [6,26]. On Sysmex analyzers, the WPC channel scattergram in some cryoglobulinemia specimens shows a unique abnormal pattern (Figure 3) and triggers an “abnormal lymphocyte” flag [27]. Studies show that leukocyte counts in cryoglobulinemia patients rise as the temperature falls and normalize after 37 °C incubation, indicating that the interference depends primarily on temperature and aggregate size, rather than the quantity or type of cryoglobulins [6,53].

**Figure 4:**
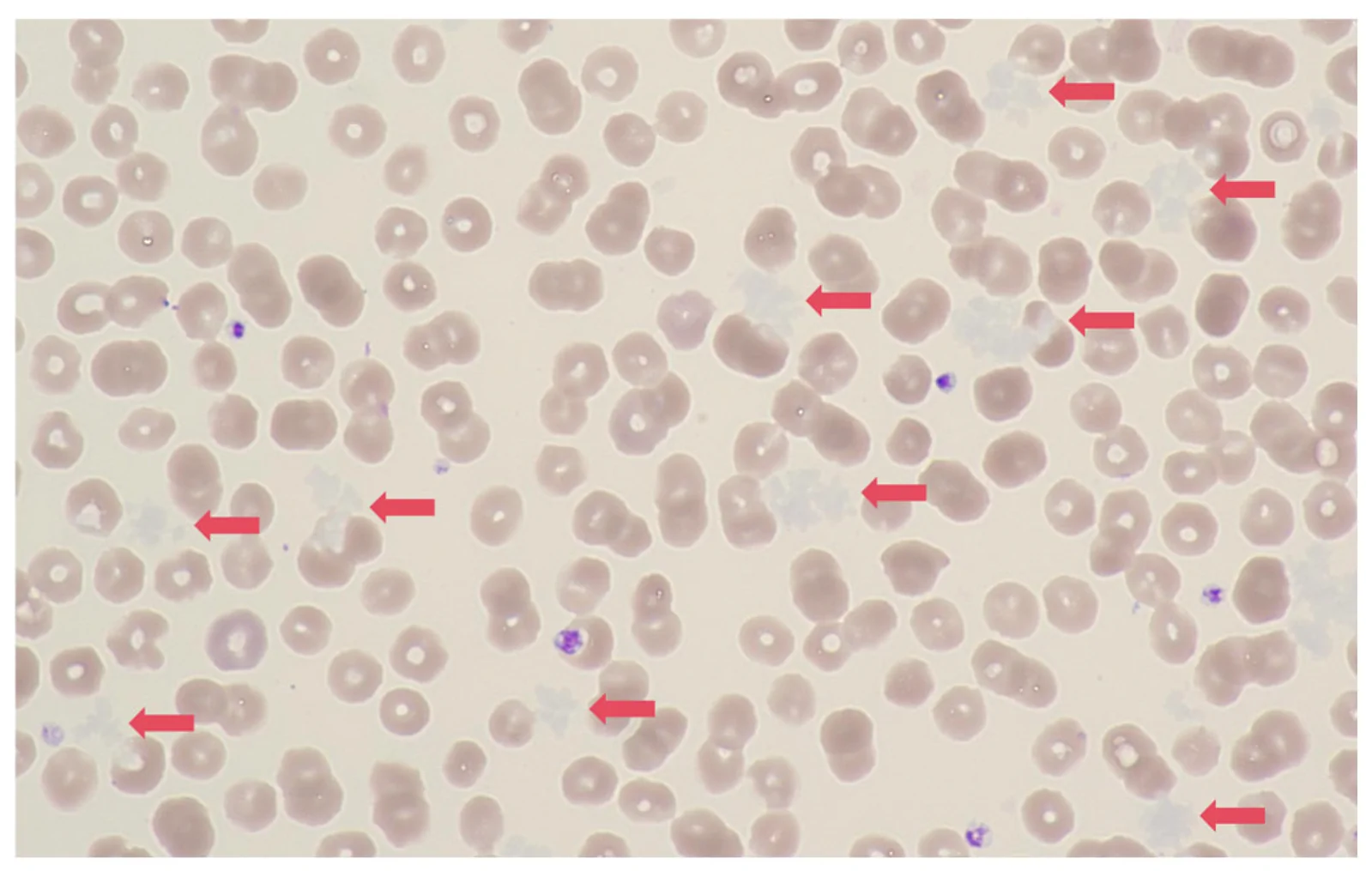
Cryoglobulin precipitates on peripheral blood smear (Wright–Giemsa stain, ×1000). Red arrows indicate amorphous cryoglobulin precipitates.

When such scattergram abnormalities and associated flags appear, cryoglobulin interference should be considered. Verification includes observing white precipitates after low-temperature storage or detecting amorphous precipitates on blood smear microscopy (Figure 4). Corrective measures include 37 °C incubation, recollecting blood with warm transport, or plasma replacement using warm isotonic diluents [1,6]. Cryofibrinogenemia similarly causes spuriously high leukocyte counts, corrected primarily by 37 °C incubation [1,6]. Paraprotein-containing specimens can also interfere with leukocyte analysis. In multiple myeloma, large amounts of plasma immunoglobulins may cause spuriously high leukocyte and platelet counts, which normalize after plasma replacement and then agree with manual counts [1,28].

#### 3.2.5. Lipids and Adipose Tissue

Hyperlipidemia is a lipid metabolism disorder characterized by elevated plasma total cholesterol and/or triglycerides. Increased plasma turbidity often interferes with HGB, MCH, and MCHC determination. Severe lipemia can also cause spuriously high leukocyte and platelet counts [5,6]. The interference mechanism involves numerous lipid particles generating persistent background scatter that masks leukocyte light scatter signals, exceeding the analyzer’s resolution and resulting in classification failure or inaccuracy. The interference intensifies with worsening lipemia [23,29,54].

**Figure 5:**
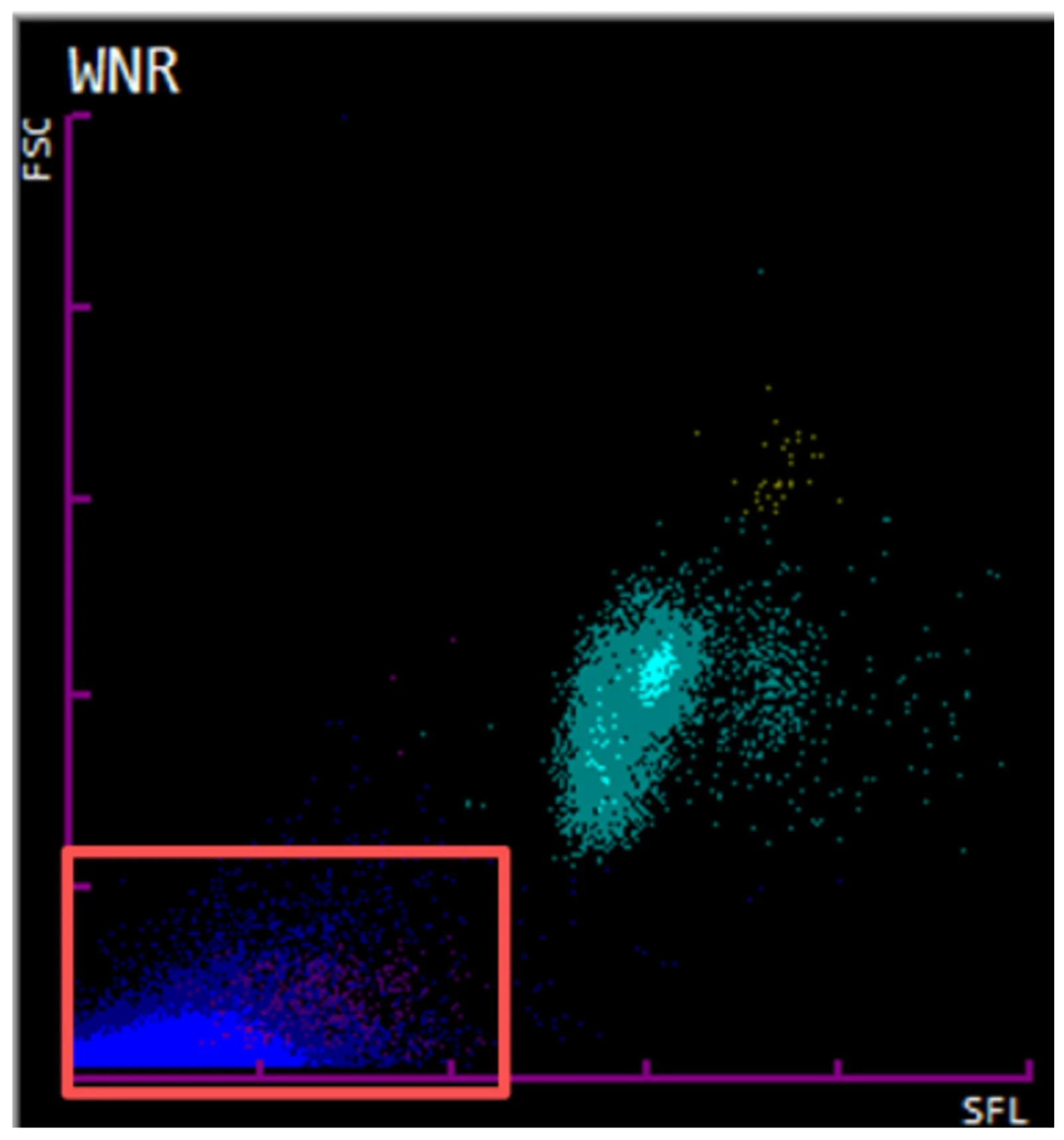
Lipid particle interference on the WNR scattergram of the Sysmex XN-9000. A high-density abnormal cluster of dark blue and purple dots is present in the lower left area of the WNR scattergram (red frame), indicative of lipid particle interference.

An in vitro experiment showed that fat emulsion can interfere with leukocyte number and morphology. After fat emulsion infusion, VCS analyzers demonstrated a decreased neutrophil percentage and increased monocyte percentage. The neutrophil population partially shifted toward and overlapped the monocyte area on the VCS scattergram, preventing accurate classification. Morphological examination revealed neutrophil swelling, degranulation, and cytoplasmic vacuolation [29]. On Sysmex analyzers, numerous lipid particles form a high-density abnormal dot cluster on the WNR scattergram (Figure 5) and trigger a “chyle/hemoglobin interference” flag. Lipemic interference can also be identified by post-centrifugation milky, turbid plasma and abnormal erythrocyte parameter ratios [1]. Corrective measures include sample dilution or plasma replacement [1,5]. For severe lipemia, a manual differential count on a blood smear is required, and the report should be labeled as a “lipemic sample.”

Adipose tissue contamination can also interfere with leukocyte analysis. Improper femoral vein puncture introduces subcutaneous adipose tissue into the specimen, causing spuriously high leukocyte counts and misclassification [30]. Blood smear microscopy reveals numerous fat droplets that stain orange-red with Sudan III. Correction requires proper blood collection and retesting.

#### 3.2.6. Microorganisms and Parasites

Extremely high concentrations of bacteria or fungi in blood can cause spuriously high leukocyte and platelet counts. This may be observed in bloodstream infections or specimen contamination [1]. Individual bacteria, especially extracellular species, may only elevate platelet counts. Microbial aggregates or large fungal spores can then interfere with leukocyte count and differential [5,6,31]. In vitro, high concentrations of bacteria do not significantly affect leukocyte detection. Fungi, particularly larger yeasts, can significantly interfere with leukocyte counts even at relatively low concentrations. The degree of interference is species-dependent, presumably related to microbial volume. The ADVIA 120/2120, Sysmex XE-2100, and Coulter LH750 analyzers all fail to recognize yeasts and misclassify them as different leukocyte types [55].

When bacteria or fungi are suspected in a specimen, peripheral blood smear microscopy confirms their presence. The source is assessed alongside clinical and other laboratory findings. Specimen contamination necessitates re-collecting blood with proper technique. To obtain accurate results, manual leukocyte counting using a hemocytometer and manual differential counting on a blood smear can be performed for confirmed bloodstream infection [1,31]. Representative cases of microbial interference remain to be accumulated, and their clinical characteristics and diagnostic clues await further study.

Malaria parasites are the most frequently reported blood parasites interfering with leukocyte analysis. Infected erythrocytes, incompletely lysed debris, large parasite bodies, or aggregates are all miscounted as leukocytes, yielding spuriously high counts. Hemozoin produced during intra-erythrocytic development is phagocytosed by neutrophils or monocytes, altering their optical properties. On Sysmex DIFF scattergrams, this appears as graying or overlapping of eosinophil and neutrophil populations, causing spurious eosinophilia [32,33,34]. To obtain reliable results, manual leukocyte counting using a hemocytometer and manual differential counting on a blood smear are required.

#### 3.2.7. Megakaryocytes and Non-Hematopoietic Cells

Megakaryocytes are rarely present in the peripheral blood under normal conditions. However, in pathological states such as myeloproliferative neoplasms (MPNs), MDS, and acute megakaryocytic leukemia, large numbers of megakaryocytes and their fragments may enter the peripheral blood. Because their volume is similar to that of leukocytes, impedance analyzers may miscount them as leukocytes [1]. There are reports showing that megakaryocyte fragments in the peripheral blood were misidentified as lymphocytes by Sysmex analyzers. On the WDF scattergram, a small number of abnormal dots appeared above the lymphocyte and monocyte areas, with high-density dots below the lymphocyte area on the side, and the analyzer triggered “blast/abnormal lymphocyte?”, “nucleated red blood cells present”, and “abnormal WBC scattergram” flags [35]. A correction formula has been proposed for cases with abundant megakaryocytes but requires further validation [1]. Therefore, when numerous megakaryocytes are observed microscopically, the WBC count should be corrected using: corrected WBC = automated WBC × 100/(100 + megakaryocytes per 100 WBCs), and the report should note “megakaryocyte interference.”

Non-hematopoietic cells, mainly carcinoma cells, are extremely rare in peripheral blood. They originate from shed solid tumors or hematological malignancies. Their variable size and morphology prevent accurate analyzer identification, leading to spuriously high leukocyte counts. A similar correction formula may be applied for non-hematopoietic cells, but further validation is required [1].

## 4. Misclassification Caused by Leukocyte Morphological and Developmental Abnormalities

Automated analyzers have inherent limits in cell recognition. When leukocytes undergo morphological changes, developmental abnormalities, or alterations in cellular contents, or when rare cell types appear, their detection characteristics often fall outside the standard classification range or overlap with those of common cells. The analyzer then struggles to identify them, tending to assign them to the population with the closest characteristics, causing failure to classify or misclassification. Abnormal scattergrams with analyzer flags offer clues for morphological review. Manual blood smear microscopy is key to confirming results. Misclassification of neutrophils and lymphocytes is relatively rare, and typical scenarios were covered in Part One. This part addresses common causes and identification when other cells are misclassified as monocytes, eosinophils, or basophils. An overview of these misclassification scenarios is provided in Table 3.

### 4.1. Common Causes of Misclassification as Monocytes

#### 4.1.1. Immature Granulocytes

In acute infection, inflammation, or stress, large numbers of immature granulocytes enter the peripheral blood. They are larger than mature granulocytes, with reduced segmentation, and their size and light scatter characteristics resemble those of monocytes, predisposing them to misclassification as monocytes [5,56]. On optically based analyzers such as Sysmex and Mindray, the presence of granulocytes at different maturation stages may result in less clearly defined borders between cell populations in the scattergram (Figure S8) [57], and the analyzer may trigger an “abnormal WBC scattergram” flag.

#### 4.1.2. Reactive Lymphocytes and Large Lymphocytes

Viral infection, drug hypersensitivity, or vaccination may stimulate the immune system to produce reactive lymphocytes. Monocytoid reactive lymphocytes are large, with irregular nuclei, abundant cytoplasm, and high nucleic acid content. They are therefore prone to misclassification as monocytes on analyzers employing semiconductor laser scatter and fluorescence staining. The differential scattergram may show upward extension of the lymphocyte population or partial fusion with the monocyte population, potentially triggering an “atypical lymphocyte” flag [58]. Large lymphocytes with volumes approaching those of monocytes are also frequently miscategorized as monocytes due to limited analyzer resolution [59].

#### 4.1.3. Dysplastic and Morphologically Abnormal Neutrophils

Dysplastic and morphologically abnormal neutrophils are seen in MDS, after radiotherapy, or during chemotherapy. They differ markedly from normal neutrophils in cell size, nuclear segmentation, and cytoplasmic granulation. Morphologically abnormal neutrophils in peripheral blood have been misclassified as monocytes by Sysmex analyzers. Microscopy confirmed these misclassified cells were neutrophils with increased volume, defective nuclear segmentation, reduced or absent granules, asynchronous nucleocytoplasmic development, and cytoplasmic vacuolation [60]. Neutrophils with morphological abnormalities caused by gene mutations, characterized by hypogranular cytoplasm and nuclear hyposegmentation, may also be miscounted as monocytes by Sysmex analyzers, often triggering “monocytosis” and “immature granulocyte” flags [61].

#### 4.1.4. Leukemic Cells

Leukemic cells have markedly diverse and abnormal morphology, hindering correct identification by analyzers. Most are misclassified into the monocyte population. Their scattergrams deviate significantly from normal patterns across analyzers with different principles [62,63,64]. On the WDF scattergram of Sysmex analyzers, acute myeloid leukemia (AML)-M2 may show numerous unclassified cells covering the monocyte and lymphocyte populations and extending upward, or poorly defined boundaries and an upward-shifted monocyte population (Figure S9A) [62]. AML-M3 may show all populations except lymphocytes merging into a large unclassifiable gray cluster, forming a broad-based, pointed teardrop shape (Figure S9B) [62]. Alternatively, AML-M3 may show a leftward shift in the immature granulocyte population merging with the monocyte population, with a clear tangent line between them (Figure S9C). Acute lymphoblastic leukemia (ALL) may show increased lymphocyte dots extending into the monocyte region, with the lymphocyte population shifting upward (Figure S9D) [62]. This misclassification likely occurs because analyzers typically assign cells with large volume, high nucleic acid content, and few granules to the monocyte population. Blasts or immature leukemic cells are therefore highly prone to being erroneously identified as monocytes.

#### 4.1.5. Hairy Cells and Lymphoma Cells

Hairy cells can be seen in hairy cell leukemia and its variant form. Numerous cases have been reported in which hairy cells were misclassified as monocytes by analyzers with different principles [65,66,67,68]. However, the identification accuracy varies among analyzers based on different principles. Analyzers relying on cellular physical properties, such as those from Beckman Coulter, tend to misclassify hairy cells into the monocyte region, leading to falsely elevated monocyte counts [65]. Analyzers employing peroxidase staining, such as the ADVIA 2120, are relatively reliable and often assign them to the large unstained cell (LUC) population [66]. On analyzers based on fluorescence staining and light scatter characteristics, such as Sysmex, the WDF and WNR scattergrams may show abnormal monocyte or lymphocyte clusters. However, the WPC channel shows no abnormal dots in the monocyte region, suggesting that this channel does not misclassify hairy cells as monocytes [65,67]. Lymphoma cells can also be misclassified as monocytes due to malignant features such as increased cell volume, altered internal structure, and abnormal nucleocytoplasmic ratio [69].

#### 4.1.6. Peroxidase-Deficient Cells

The Siemens ADVIA 2120 analyzer classifies leukocytes based on cellular physical properties and peroxidase staining. The staining intensity varies among different cell populations: eosinophils are strongly positive, neutrophils are positive, monocytes are weakly positive, and lymphocytes are essentially negative. However, abnormal granulocyte peroxidase activity can cause population shifts and classification errors. When peroxidase is partially deficient, or its activity is reduced, the staining intensity of granulocytes approaches that of monocytes. These cells are readily misclassified into the monocyte population (Figure S10A,B) [60], causing spuriously elevated monocyte counts and underestimated neutrophil and eosinophil counts. When peroxidase is completely deficient or its activity is lost, these cells are assigned to the LUC population [70].

### 4.2. Common Causes of Misclassification as Eosinophils

#### 4.2.1. Neutrophils Containing Abnormal Granules

In severe stress responses, particularly acute bacterial infections, coarse toxic granules form within the cytoplasm of neutrophils. Such cells may be erroneously identified as eosinophils by Sysmex analyzers. The WDF scattergram shows a rightward shift in the neutrophil population with poorly defined boundaries against the eosinophil population, and the analyzer may trigger “abnormal WBC scattergram” and “eosinophilia” flags [71]. Granulocyte colony-stimulating factor (G-CSF)-induced neutrophil morphological changes are well recognized, but the misclassification of morphologically altered neutrophils as eosinophils by Sysmex analyzers is rarely reported. In a Chinese case series, two patients receiving pegylated recombinant human G-CSF (PEG-rhG-CSF) developed coarse, unevenly sized, irregularly distributed orange-red granules in some neutrophils. On the WDF scattergram, eosinophil red dots deviated and overlapped with the neutrophil population, causing spuriously elevated eosinophil counts [72]. The mechanism is similar in both situations. The abnormal granules increase the internal complexity of neutrophils, enhancing the side scatter (SSC) signal, which shifts the cell population and causes misclassification [71,72].

#### 4.2.2. Neutrophils Engulfing Platelets or Other Substances

Neutrophils phagocytosing platelets accompanied by platelet satellitism can spuriously elevate eosinophil counts on Sysmex analyzers [73,74]. The likely mechanism is that phagocytosed or adherent platelets increase cellular granularity and complexity, shifting the neutrophil population rightward on the WDF scattergram and overlapping it with the eosinophil population, leading to misclassification [73]. In malaria, neutrophils or monocytes engulfing hemozoin may be similarly misclassified (see Section 3.2.6).

#### 4.2.3. Myeloid Neoplastic Cells

**Figure 6:**
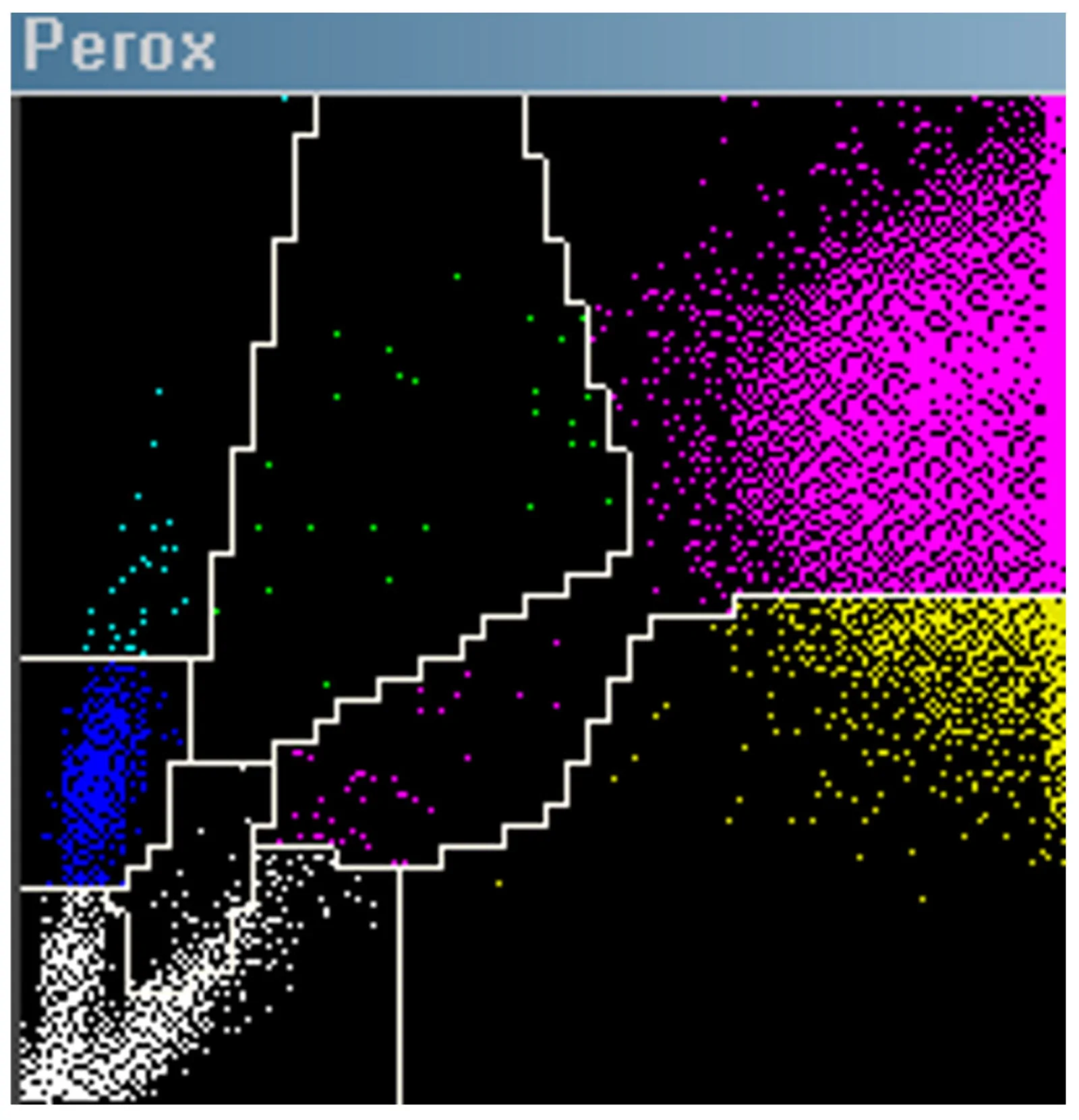
Abnormal promyelocytes misclassified as eosinophils on the Perox scattergram of the Siemens ADVIA 2120. The Perox scattergram shows inseparable neutrophil (represented by purple dots) and eosinophil (represented by yellow dots) populations shifted en bloc to the right into the strongly MPO-positive region.

Acquired gene mutations in hematopoietic stem/progenitor cells can produce myeloid neoplastic cells with markedly abnormal morphology and function. Some are misclassified as eosinophils due to overlapping detection characteristics. On the ADVIA 2120 analyzer, abnormal promyelocytes in acute promyelocytic leukemia (APL) specimens are often misclassified into the eosinophil population. The Perox scattergram shows inseparable neutrophil and eosinophil populations shifting rightward into the strongly myeloperoxidase (MPO)-positive region (Figure 6). The reason for this misclassification may be that abnormal promyelocytes contain abundant azurophilic granules rich in MPO in their cytoplasm [75]. Spuriously elevated eosinophil counts also occur in AML with t(8;21) and MDS, where microscopy reveals no true eosinophilia but instead hypogranular, hyposegmented neutrophils and blasts with Auer rods [75]. In AML-M4, blasts with granules and crystalline inclusions may also be misclassified as eosinophils [76]. These cells may share overlapping characteristics with eosinophils in terms of cell volume, granular MPO activity, or cellular complexity, leading to misclassification by the analyzer.

#### 4.2.4. Hypogranular Eosinophils

In MPN, MDS, and severe infections, eosinophils may exhibit granule reduction, degranulation, and vacuolation. Hypogranular eosinophils can be misclassified as neutrophils by Sysmex XN-20 and Beckman Coulter DxH 800 analyzers. The mechanism is that granule reduction attenuates light scatter signals, making it difficult for the analyzer to distinguish them from neutrophils [77,78]. On the leukocyte differential scattergram, the eosinophil population shifts leftward and overlaps extensively with neutrophils, yielding spuriously low eosinophil and spuriously high neutrophil counts.

### 4.3. Common Causes of Misclassification as Basophils

#### 4.3.1. Reactive Lymphocytes

Reactive lymphocytes exhibit diverse morphological features and represent a weak point in cell recognition and classification by analyzers. They can be misidentified as monocytes (see Section 4.1.2) or basophils [60,79,80,81]. In dengue fever patients, spuriously elevated basophil counts have been reported. The analyzer may trigger “atypical lymphocyte” and “basophilia” flags, but smear microscopy confirms these cells are reactive lymphocytes with basophilic cytoplasm [80]. This misclassification likely results from their resistance to BASO channel reagents.

#### 4.3.2. Lymphoid Neoplastic Cells

Various lymphoid neoplastic cells can spuriously elevate basophil counts across analyzers with different principles [82,83,84]. In CLL and Sézary syndrome, abnormal lymphocytes may be misclassified as basophils by Sysmex analyzers. The analyzers triggered “abnormal lymphocyte” and “Blasts” flags, but microscopy confirmed morphologically abnormal lymphocytes [82]. On the ADVIA 2120, “flower cells” from a patient with acute-phase adult T-lymphoma leukemia (ATLL) were misclassified as basophils. The BASO scattergram showed increased dots in the basophil area, and the Perox scattergram showed increased dots in the LUC area [83]. Similar phenomena have been observed on this analyzer in various lymphoid malignancies, including CLL, B-cell lymphoma, T-cell lymphoma, and ALL. Abnormal cells appear with various scatter patterns in the basophil counting area of the BASO scattergram [84]. The likely mechanism is that these abnormal cells resist the acidic hemolytic reagent and surfactant in the BASO channel and are consequently miscounted as basophils [83,84].

#### 4.3.3. Myeloma Cells

Myeloma cells can be misclassified as basophils or monocytes by different analyzers [5,81,85]. The literature reports a case of multiple myeloma (MM) with a high proportion of myeloma cells in the peripheral blood. The ADVIA 120 misclassified them as basophils, whereas the SE-9000 misclassified them as monocytes [81]. However, not all myeloma cells are misclassified. In another case of relapsed MM, the ADVIA 2120 BASO scattergram showed increased dots in the blast region, and the Perox scattergram showed increased LUCs. These cells were confirmed as myeloma cells by smear microscopy [86]. These cases suggest that membrane or cytoplasmic properties vary among myeloma cells, affecting analyzer classification.

## 5. Analytical Errors Caused by Preanalytical and Systematic Factors

Preanalytical and systematic factors represent another important source of inaccuracy in leukocyte analysis. The reliability of test results rests on standardized preanalytical procedures, along with systematic factors such as instrument performance, reagent efficacy, and laboratory environmental conditions. Deviation in any aspect can compromise leukocyte count and differential. The effects of these factors and corresponding preventive measures are described below.

### 5.1. Preanalytical Factors

Preanalytical sample handling begins with patient preparation and extends through specimen collection, transport, storage, and the period before analysis. The type of disinfectant used at the venipuncture site, the blood collection procedure, anticoagulant usage, specimen transport, and specimen storage time can all affect leukocyte count and differential [5,6,87,88,89,90,91,92,93,94].

#### 5.1.1. Sample Collection

Fibrin strand formation, inappropriate blood collection volume, specimen contamination, and improper anticoagulant usage can all affect leukocyte analysis.

Fibrin strands often result from long-term indwelling central venous catheters or difficult venipuncture and can enter the sample during collection. Such specimens cause spuriously elevated leukocyte counts on Coulter-based analyzers. The histogram shows a steep ascending limb and a sloping descending limb. Fibrin strands can also block the counting aperture, causing counting failure [1,88]. On Sysmex analyzers, fibrin strands may produce abnormal dots on the WDF scattergram (Figure S11A), causing the WDF channel leukocyte count to be significantly higher than the WNR channel count. Smear microscopy reveals fibrin strands admixed with leukocytes (Figure S11B).

Excessive blood collection causes inadequate anticoagulant mixing or insufficient anticoagulant ratio, leading to microclot formation and interference with leukocyte counting [5,6]. Insufficient blood collection results in relative EDTA-K_2_ excess that directly destroys leukocytes [5]. Erroneous use of other anticoagulants alters cell morphology and size, interfering with counting and classification.

Specimen contamination is a common preanalytical error. Exogenous fluids or therapeutic drugs introduced by drawing blood from the same side as an intravenous infusion can affect CBC results through dilution or direct interference. Buonocore et al. found that glucose contamination caused spuriously low total leukocyte counts and abnormal differentials on analyzers based on both laser scatter combined with peroxidase staining and laser scatter combined with fluorescent nucleic acid staining [89].

#### 5.1.2. Sample Storage and Transport

Leukocytes undergo degenerative changes over time after blood collection. These morphological changes alter their physical characteristics and can interfere with cell recognition and classification by analyzers of different principles. Therefore, specimen storage time directly affects test results [5,90]. Several studies have comprehensively assessed the impact of storage time on leukocyte differential across analyzers with different principles [90,91,92,93,94]. Although the direction and magnitude of bias vary by leukocyte type, all indicate that longer storage causes greater scattergram dispersion and classification deviation. Conversely, insufficient storage may cause misclassification because the anticoagulant effect has not stabilized. Vigorous shaking during transport or excessive mixing before analysis can mechanically damage leukocytes and alter morphology, affecting results [90].

To minimize preanalytical errors, we recommend standardized blood collection, strict transport compliance, and testing as soon as possible after the anticoagulant effect stabilizes.

### 5.2. Systematic Factors

Beyond preanalytical factors, instrument performance, reagent efficacy, calibration and quality control, and environmental conditions also affect leukocyte analysis accuracy.

#### 5.2.1. Instrument

Abnormal scattergrams or flags warrant systematic investigation of analyzer performance and operational status. Analyzers have limited reproducibility for low-prevalence cell populations in peripheral blood, such as monocytes and basophils, and their accuracy shows relatively poor correlation with reference methods [5,95,96,97]. At the hardware level, any abnormality can cause leukocyte misclassification, ranging from blocked or contaminated sample lines, dirty reaction chambers, and inadequate mixing, to aging detection-unit lasers, unstable sheath-flow pressure, and signal processing circuit board failures. When relevant flags appear, the analyzer should be taken out of service and these hardware faults investigated.

#### 5.2.2. Reagents

Reagent efficacy directly affects leukocyte differential accuracy. Improper storage or expired lytic reagents, diluents, staining solutions, or cleaning solutions cause classification errors. Strict adherence to storage requirements, standardized bottle-opening and expiry management, and inter-lot quality control monitoring can effectively prevent such errors.

#### 5.2.3. Quality Control (QC), Calibration, and Maintenance

Calibration drift, uncorrected QC failures, and absent preventive maintenance introduce systematic errors that are difficult to detect immediately and persistently compromise leukocyte analysis reliability. Strict implementation of standardized procedures covering calibration, QC, and maintenance is therefore key to preventing these errors.

#### 5.2.4. Environment

Uncontrolled laboratory temperature and humidity also affect leukocyte analysis. Low temperature can cause fine crystal precipitation from the diluent, affecting cell morphology and reducing lytic reagent efficacy, leading to incomplete erythrocyte lysis. Excessively high temperature or humidity also interferes with differential. Maintaining constant temperature and humidity, cleanliness, stable voltage, and vibration protection is essential for ensuring accurate and reliable leukocyte differential results.

## 6. Other Factors Affecting Leukocyte Analysis

### 6.1. High and Low Leukocyte Counts

In specimens with excessively high or low total leukocyte counts, counting inaccuracies and classification failure are common, with specific interference thresholds varying by detection system principle and performance. In high-count specimens, multiple cells pass through the detection aperture or laser beam simultaneously, causing signal overlap. Excessive leukocytes consume large amounts of lytic reagent, leading to incomplete erythrocyte lysis and inadequate leukocyte processing. Leukocyte numbers exceeding the analyzer’s linear range further contribute to classification failure or unreliable results. In low-count specimens, insufficient and unevenly distributed cells in the counting chamber markedly reduce differential reproducibility and accuracy, often resulting in classification failure. Blood cell morphology analysis systems can rapidly and accurately identify cells in leukopenic specimens, serving as an effective supplement to manual microscopy [98]. Therefore, both excessively high and low leukocyte count specimens require manual microscopy or validated alternative techniques to obtain reliable differential results.

### 6.2. Abnormal Hemoglobin

**Figure 7:**
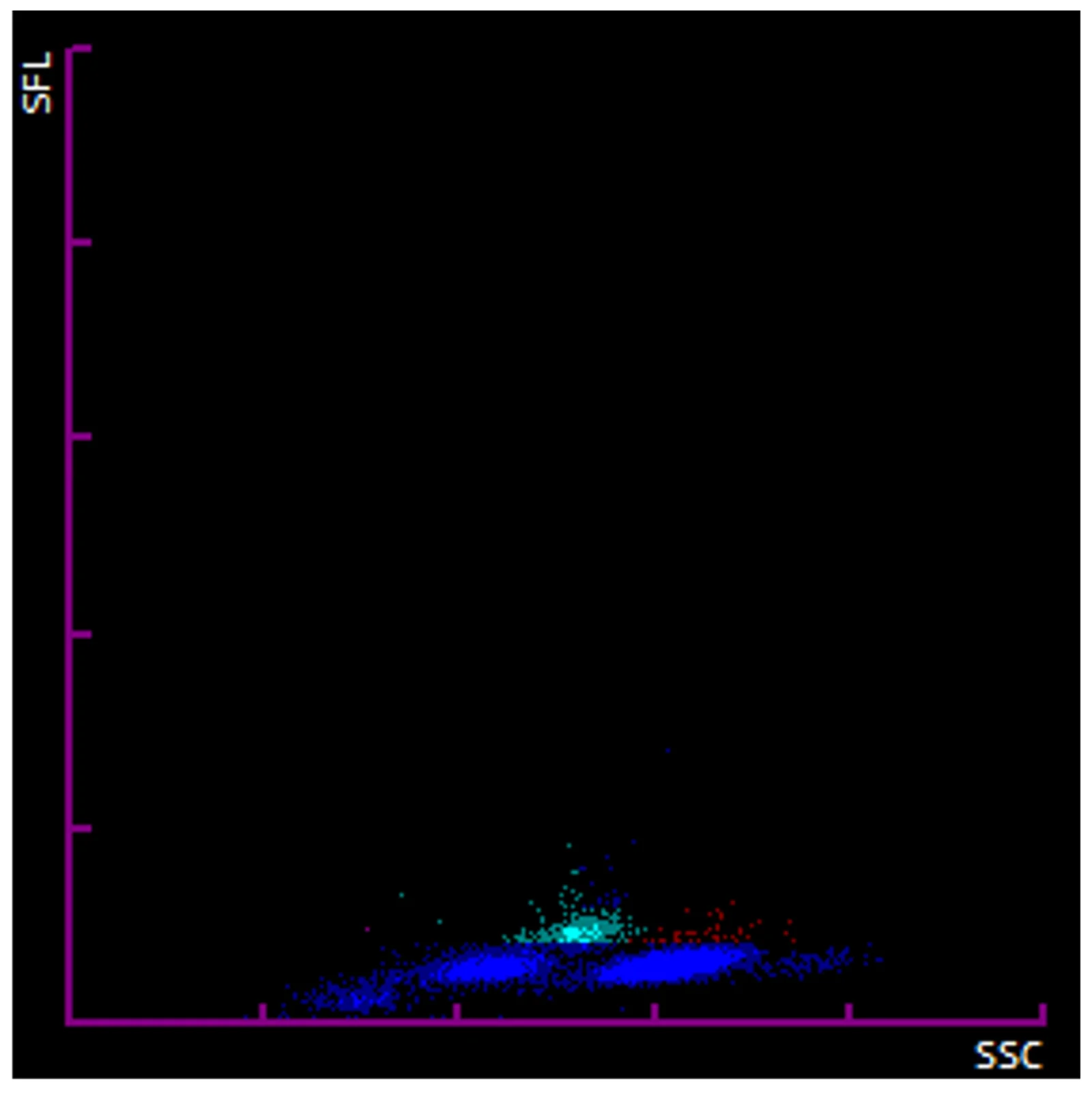
Abnormal hemoglobin interference on the WDF scattergram of the Sysmex XN-9000. Decreased fluorescence signals are observed across all leukocyte populations. This pattern interferes with leukocyte count and differential.

Abnormal hemoglobins, such as methemoglobin and certain globin gene mutation products, interfere with leukocyte differential on optically based analyzers, including Sysmex and Mindray [99,100,101,102,103,104,105]. DIFF or WDF scattergrams show generally low fluorescence signals across cell clusters, producing a flattened appearance (Figure 7) and often triggering an “abnormal WBC scattergram” flag. The mechanism is incompletely understood. It may involve abnormal hemoglobin released from erythrocytes lysed in the DIFF or WDF chamber hindering fluorescent dye staining of leukocyte nucleic acids [99]. Some investigators propose that the fluorescent dye exhibits higher affinity for certain abnormal hemoglobins than for nucleic acids, or that these abnormal hemoglobins reduce dye permeability across the leukocyte membrane [100,101,102]. Interference diminishes as abnormal hemoglobin concentration decreases [99]. The WNR channel of the same analyzer is usually unaffected, possibly due to its lytic reagent potency or dye properties. Such specimens may still display normal distribution in the Perox channel of the ADVIA 2120 [104]. Corrective measures include manual differential counting or switching to an analytical system with a different detection principle.

## 7. Conclusions

This review categorizes the diverse error sources affecting automated leukocyte analysis. For severe interferences, manual methods are the ultimate approach for obtaining reliable results. However, it must be noted: blood smear review is used for morphological confirmation and accurate differential counts but cannot yield an accurate total leukocyte count, whereas manual counting with a hemocytometer provides a reliable total count, but it is labor-intensive and highly subjective.

In comparison with previous reviews, we synthesized the diverse causes of unreliable leukocyte results across the analytical pipeline. Aligning with the practical investigative pathways of clinical laboratory professionals, we established a practical diagnostic workflow from anomaly recognition to result reporting. Incorporating both legacy and current systems, we compared the scattergram and histogram patterns of various interferents across platforms with distinct detection principles to broaden applicability to diverse clinical laboratories. We summarized a comprehensive range of corrective measures and explicitly distinguished routine clinical practices from case-based or experimental methods. This provides laboratory professionals with broader corrective options when routine methods prove ineffective.

Currently, resolving complex interferences still relies heavily on the empirical judgment of laboratory professionals. Although automated digital morphology systems have become important adjuncts to blood smear review, they cannot yet meet the screening demands of high-volume specimens. Therefore, continuous upgrades of analyzers remain the fundamental direction, focusing on enhancing anti-interference performance, improving the recognition of complex cell populations, and enriching scattergram information. The integration of artificial intelligence represents a pivotal trend in automated hematology analysis. Existing studies demonstrate that deep learning models can mine the distribution patterns of abnormal cell clusters in scattergrams and integrate CBC digital parameters to achieve rapid classification of hematolymphoid malignancies and efficient screening for APL [106,107]. This also enables the transition of analyzer flags from isolated single-parameter flags to intelligent disease flags. Although prospective validation is still required, accumulating studies highlight the immense potential of artificial intelligence in advancing intelligent diagnostic systems.

## Figures and Tables

**Table 1:** Spurious low leukocyte counts combined with leukocyte misclassification.

Interfering Factor	Histogram/Scattergram Features on Analyzers with Different Principles	Corrective Measures
EDTA-dependent neutrophil agglutination	**Impedance:** Dense dots appear above the area of PMN (top of the WBC graph), or it is difficult to separate WBC classes, generating flags such as “immature granulocyte” or “band cells” [4,5,6,7]. **Laser scatter combined with peroxidase staining:** A band of dots may appear in the upper right corner of the leukocyte differential scattergram, triggering a high peroxidase content flag [5,6]. **VCS technology:** The neutrophil population shifts markedly toward and overlaps the monocyte region, with markedly increased MNV and NDW [8].	A: Use citrate anticoagulant and multiply the leukocyte count and related parameters by 1.1 for correction [1,9]. (Note: leukocyte aggregation may also occur with sodium citrate or heparin [6].) B: Add kanamycin to the sample and reanalyze [1,10]. B: Vortex the EDTA-anticoagulated blood sample for 1–2 min before analysis [1]. B: Collect fingerstick blood and immediately test using the predilution mode [6,11]. B: Collect blood samples using anticoagulants such as CTAD or CPT [1,4].
Temperature-mediated neutrophil agglutination	Scattergram patterns are similar to those of EDTA-dependent neutrophil agglutination (see above). In addition, the WBC histogram on impedance analyzers may show a T2 flag [12] or abnormal distribution of granulocytes and monocytes [13], and VCS parameters (MNV and NDW) are markedly elevated.	A: Incubate the EDTA-anticoagulated blood sample at 37 °C for 10–20 min and retest [1,12,14]. B: Treat plasma with anti-IgM antibody or DTT [6]. B: Perform plasma replacement [14]. B: Test immediately after collection of fingerstick blood [12].
Lymphocyte agglutination	**Based on cell volume combined with peroxidase staining (Technicon H6000):** an abnormal dot band appears above the monocyte–basophil area on the Perox scattergram [15].	B: Switch to alternative anticoagulants (sodium citrate or heparin) [5,6,16]. B: Immediate dilution and testing of fingerstick blood [5,6].
Plasma interferents	**Sheath-flow impedance combined with semiconductor laser flow cytometry:** A significant discrepancy is observed between WDF and WNR channel leukocyte counts. The WNR scattergram shows numerous interfering dots near the *X*-axis or a reduction in dots in the leukocyte counting area [17].	B: If the smear estimate matches the WDF result, accept the WDF result [18]. B: Use analyzers based on other principles [18]. B: Use predilution mode [19].

**Note:** Evidence categories were assigned separately to each corrective measure based on the strength and applicability of the supporting literature. **Category A,** established and validated routine practice—widely accepted and suitable for routine laboratory use, typically supported by guidelines, consensus, systematic reviews, or prospective multicenter studies. **Category B,** experimental or case-based methods not widely accepted—supported only by individual case reports or small early studies; may be attempted when Category A methods fail. Existing professional guidelines (e.g., ICSH, CLSI) lack specific consensus recommendations for most corrective procedures. Therefore, where no guideline specifically addresses a given interference factor, the category reflects the highest level of available primary literature. **Abbreviations:** PMN, polymorphonuclear neutrophil; WBC, white blood cell; MNV, mean neutrophil volume; NDW, neutrophil distribution width; CTAD, citrate–theophylline–adenosine–dipyridamole; CPT, citrate-pyridoxal 5′-phosphate-tris; DTT, dithiothreitol; WDF, white cell differential channel; WNR, white cell nucleated red channel; VCS, volume, conductivity, and scatter.

**Table 2:** Spurious high leukocyte counts combined with leukocyte misclassification.

Interfering Factor	Histogram/Scattergram Features on Analyzers with Different Principles	Corrective Measures
Platelet aggregation	**Impedance:** The leukocyte histogram shows an elevated take-off at 35 fL [20]. The platelet histogram displays a steep peak, an upturned tail, or an irregular contour. **Sheath-flow impedance combined with semiconductor laser flow cytometry:** The PLT-I histogram shows a steep, unsmoothed curve or a markedly elevated tail. The PLT-F scattergram reveals enhanced FSCW dispersion. **Laser scatter combined with peroxidase staining:** On the Perox scattergram, numerous abnormal white dot clusters appear in the “noise/platelet aggregation” recognition zone, disrupting normal cell population boundaries. Some dots connect to the lymphocyte and monocyte clusters [5].	A: Switch to citrate anticoagulant and retest. Multiply the leukocyte count and related parameters by a dilution factor of 1.1 [1]. B: Vortex the specimen for 1–2 min and retest immediately. This can dissociate some platelet aggregates without disturbing other CBC parameters [1,21]. B: Keep the anticoagulated blood at 37 °C until analysis [1].
Large platelets	**Sheath-flow impedance combined with semiconductor laser flow cytometry:** Numerous dark blue dots cluster on the right side of the debris area on the WDF scattergram. On the WNR scattergram, the dots in the debris region are markedly increased and extend toward the upper right into the leukocyte area. The PLT-I curve is broadened with an irregular contour. On the PLT-F scattergram, dots extend into the larger cell volume and stronger fluorescence area, and may even border the leukocyte region.	A: For specimens severely interfered with by large platelets, to obtain accurate results, perform manual leukocyte counting using a hemocytometer and manual differential counting on a blood smear [1,5]. B: in urgent situations, blood smear-based estimation of leukocyte count may be attempted; however, this method cannot provide an accurate count and is highly subjective [5].
NRBCs	**Impedance:** The WBC histogram shows a double peak in the lymphocyte region [22]. **Laser scatter combined with peroxidase staining:** The Perox scattergram shows the NRBC population connected to the lymphocyte population and extending upward along the lymphocyte cluster [6]. **Sheath-flow impedance combined with semiconductor laser flow cytometry:** A population of NRBCs appears below the lymphocyte cluster, closely adjacent and with the same color as the lymphocytes.	A: Manually correct the leukocyte count using the formula: corrected WBC = X × 100/(100 + Y), where X is the uncorrected WBC count and Y is the number of NRBCs counted per 100 leukocytes during the differential [1].
Lysis-resistant RBCs	**VCS technology:** The lymphocyte population appears diffuse with blurred boundaries. An abnormal cluster of discrete dots appears in the lower left of this area [23]. **Sheath-flow impedance combined with semiconductor laser flow cytometry:** A significant discrepancy exists between the DIFF and BASO channel leukocyte counts. In the lower left corner of the WNR scattergram (low fluorescence and low side scatter region), an abnormal blue dot cluster or band lies tightly along the *X*-axis.	B: Dilute the blood sample and retest [1,23]. B: Increase the lytic reagent volume, prolong lysis time, or use reagents with stronger lytic capacity [6,24].
Hyperbilirubinemia	**VCS technology:** Leukocyte populations overlap and lose their boundaries, leading to differential failure or misclassification [23,25].	B: Dilute the blood sample and retest, which may reduce interference in some cases [1,23].
Cryoglobulin	**VCS technology:** An abnormal small peak with an early take-off appears at the far left of the leukocyte volume histogram [26]. **Laser scatter combined with peroxidase staining:** Abnormal interfering dots are present near the lymphocyte area or above the neutrophil area on the Perox scattergram [6,26]. **Sheath-flow impedance combined with semiconductor laser flow cytometry:** On the WPC channel SSC-SFL scattergram, a unique triangular abnormal dot cluster forms below the leukocyte population, triggering an “abnormal lymphocyte” flag [27].	A: Incubate the specimen at 37 °C for 10-20 min and retest immediately [1,6]. B: Recollect blood and transport it warm until analysis [1,6]. B: Perform plasma replacement using warm isotonic diluents maintained at 37 °C [1].
Cryofibrinogen	/	A: Incubate the specimen at 37 °C for 10-20 min and retest immediately [1,6].
Paraprotein-associated diseases	/	B: Plasma replacement [1,28].
Lipids	**VCS technology:** The scattergram shows the neutrophil population partially shifting to the monocyte area and overlapping with the monocyte population [29]. **Sheath-flow impedance combined with semiconductor laser flow cytometry:** A high-density abnormal signal cluster forms in the lower left of the WNR channel FSC/SFL scattergram.	B: Dilute the sample or perform plasma replacement [1,5].
Adipose tissue	/	A: Recollect blood and retest [1,30].
Microorganisms	/	A: For specimen contamination, properly collect a fresh blood sample and retest [1]. A: For confirmed bloodstream infection, to obtain accurate results, perform manual leukocyte counting using a hemocytometer and manual differential counting on a blood smear [1,31].
Parasites (Plasmodium)	**Sheath-flow impedance combined with semiconductor laser flow cytometry:** On the WDF scattergram, the eosinophil and neutrophil populations may appear grayish or overlap each other [32,33,34].	A: Perform manual leukocyte counting using a hemocytometer and manual differential counting on a blood smear.
Megakaryocytes and Non-Hematopoietic Cells	**Sheath-flow impedance combined with semiconductor laser flow cytometry:** On the WDF scattergram, a few abnormal dots are visible above the lymphocyte and monocyte areas, and high-density dots are present laterally below the lymphocyte area [35].	B: Manually correct the leukocyte count using the formula: corrected WBC = X × 100/(100 + Y), where X is the uncorrected WBC count, and Y is the number of megakaryocytes or non-hematopoietic cells counted per 100 leukocytes during the differential [1].

**Note:** Evidence categories are defined as in Table 1. **Category A,** established and validated routine practice; **Category B,** experimental or case-based methods not widely accepted. Each corrective measure was categorized separately. **Abbreviations:** PLT-I, platelet impedance; PLT-F, platelet fluorescence; FSCW, forward scatter width; CBC, complete blood count; WDF, white cell differential channel; WNR, white cell nucleated red channel; WBC, white blood cell; NRBC, nucleated red blood cell; Lysis-resistant RBCs, lysis-resistant red blood cells; VCS, volume, conductivity, and scatter; DIFF, differential channel; BASO, basophil channel; WPC, white progenitor cell channel; FSC, forward scatter; SSC, side scatter; SFL, side fluorescence.

**Table 3:** Common scenarios of leukocyte differential misclassification by automated hematology analyzers.

Reported Leukocyte Type	Actual Cell Type
Monocytes	Immature granulocytes, reactive lymphocytes, large lymphocytes, dysplastic and morphologically abnormal neutrophils, leukemic cells, hairy cells, lymphoma cells, peroxidase-deficient cells, etc.
Eosinophils	Neutrophils containing abnormal granules, neutrophils engulfing platelets or other substances, myeloid neoplastic cells, hypogranular eosinophils (misclassified as neutrophils), etc.
Basophils	Reactive lymphocytes, lymphoid neoplastic cells, myeloma cells, neutrophils containing toxic granules, dysplastic neutrophils, etc.

## Data Availability

No new data were created or analyzed in this study. Data sharing is not applicable to this article.

## Supplementary Materials

The following supporting information can be downloaded at: https://www.mdpi.com/article/10.3390/diagnostics16213578/s1, Figure S1: Lymphocyte aggregates on peripheral blood smear (Wright–Giemsa stain, ×1000). The smear shows abnormal lymphocyte aggregates in a patient with CLL; Figure S2: Platelet aggregate interference on the PLT-I histogram and PLT-F scattergram of the Sysmex XN-9000; Figure S3: Platelet aggregate interference on the Perox scattergram of the Siemens ADVIA 2120; Figure S4: Large platelet interference on the PLT-I histogram, PLT-F, WDF, and WNR scattergrams of the Sysmex XN-9000; Figure S5: NRBC interference on the Perox scattergram of the Siemens ADVIA 2120; Figure S6: NRBC interference on the WDF scattergram of the Sysmex XN-9000; Figure S7: Lysis-resistant RBC interference on the WNR scattergram of the Sysmex XN-9000; Figure S8: Immature granulocytes misclassified as monocytes on the WDF scattergram of the Sysmex XN-9000; Figure S9: Leukemic cells misclassified as monocytes on the WDF scattergram of the Sysmex XN-9000; Figure S10: Peroxidase-deficient neutrophils misclassified as monocytes on the Perox scattergrams of the Siemens ADVIA 2120; Figure S11: Fibrin strand interference on the WDF scattergram of the Sysmex XN-9000 and peripheral blood smear; Table S1: Chinese search terms used for CNKI; Table S2: Detection principles and characteristics of hematology analyzers discussed in this review.
