# Supplementary material for: Multidimensional Tracing of Error Sources and Structured Corrective Strategies for Leukocyte Count and Differential on Automated Hematology Analyzers
Source: Diagnostics (Basel). 2026 Oct 29;16(21):3578. doi: 10.3390/diagnostics16213578 (PMC13761757; doi:10.3390/diagnostics16213578)
Supplement: Supplementary file 1 [file diagnostics-16-03578-s001.zip › Figure S1.pdf]

Figure S1

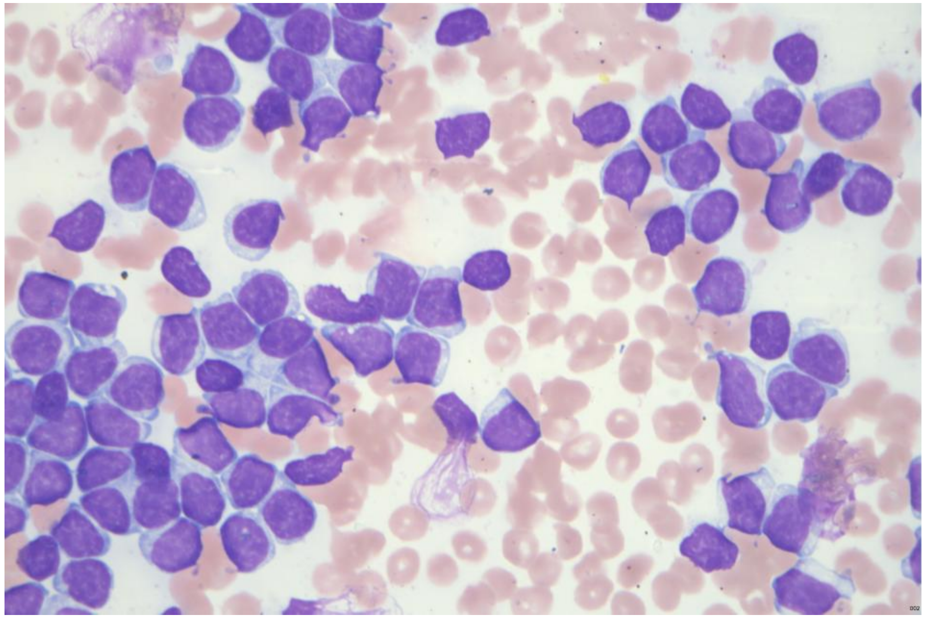

Lymphocyte aggregates on peripheral blood smear (Wright–Giemsa stain, ×1,000). The smear shows abnormal lymphocyte aggregates in a patient with CLL.
