# Supplementary material for: Multidimensional Tracing of Error Sources and Structured Corrective Strategies for Leukocyte Count and Differential on Automated Hematology Analyzers
Source: Diagnostics (Basel). 2026 Oct 29;16(21):3578. doi: 10.3390/diagnostics16213578 (PMC13761757; doi:10.3390/diagnostics16213578)
Supplement: Supplementary file 1 [file diagnostics-16-03578-s001.zip › Figure S10.pdf]

Figure S10

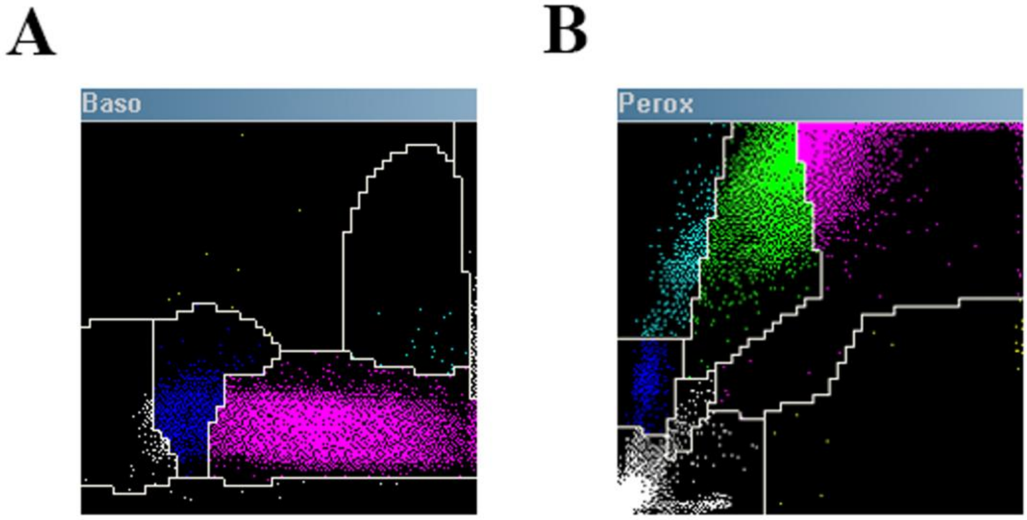

Peroxidase-deficient neutrophils misclassified as monocytes on the Perox scattergrams of the Siemens ADVIA 2120.

(A) BASO scattergram showing normal separation of mononuclear (MN) and polymorphonuclear (PMN) populations.

(B) Perox scattergram showing neutrophils with partial peroxidase deficiency shifted leftward into the monocyte population, resulting in spuriously elevated monocyte counts.
