# Supplementary material for: Multidimensional Tracing of Error Sources and Structured Corrective Strategies for Leukocyte Count and Differential on Automated Hematology Analyzers
Source: Diagnostics (Basel). 2026 Oct 29;16(21):3578. doi: 10.3390/diagnostics16213578 (PMC13761757; doi:10.3390/diagnostics16213578)
Supplement: Supplementary file 1 [file diagnostics-16-03578-s001.zip › Figure S11.pdf]

Figure S11

A

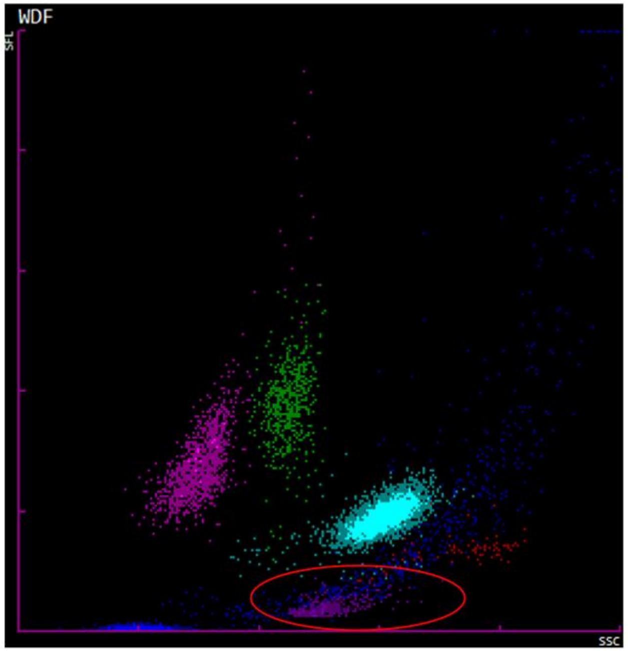

B

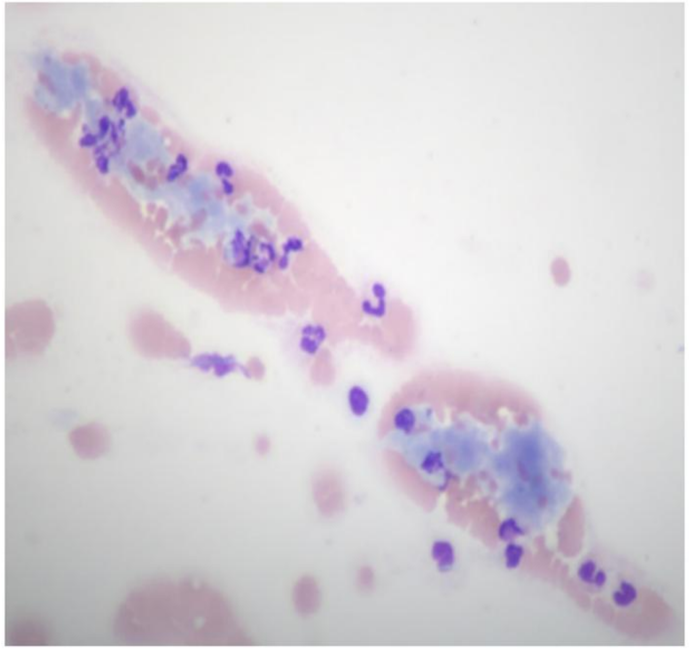

Fibrin strand interference on the WDF scattergram of the Sysmex XN-9000 and peripheral blood smear.

(A) WDF scattergram showing a dense cluster of purple dots in the lower left region of the neutrophil population, accompanied by a band of blue dots extending upward from the bottom horizontal axis and gradually becoming more diffuse; the overlapping area of purple and blue dots is circled in red.

(B) Peripheral blood smear (Wright–Giemsa stain, ×1,000) revealing fibrin strands intermingled with leukocytes.
