# Supplementary material for: Multidimensional Tracing of Error Sources and Structured Corrective Strategies for Leukocyte Count and Differential on Automated Hematology Analyzers
Source: Diagnostics (Basel). 2026 Oct 29;16(21):3578. doi: 10.3390/diagnostics16213578 (PMC13761757; doi:10.3390/diagnostics16213578)
Supplement: Supplementary file 1 [file diagnostics-16-03578-s001.zip › Figure S2.pdf]

Figure S2

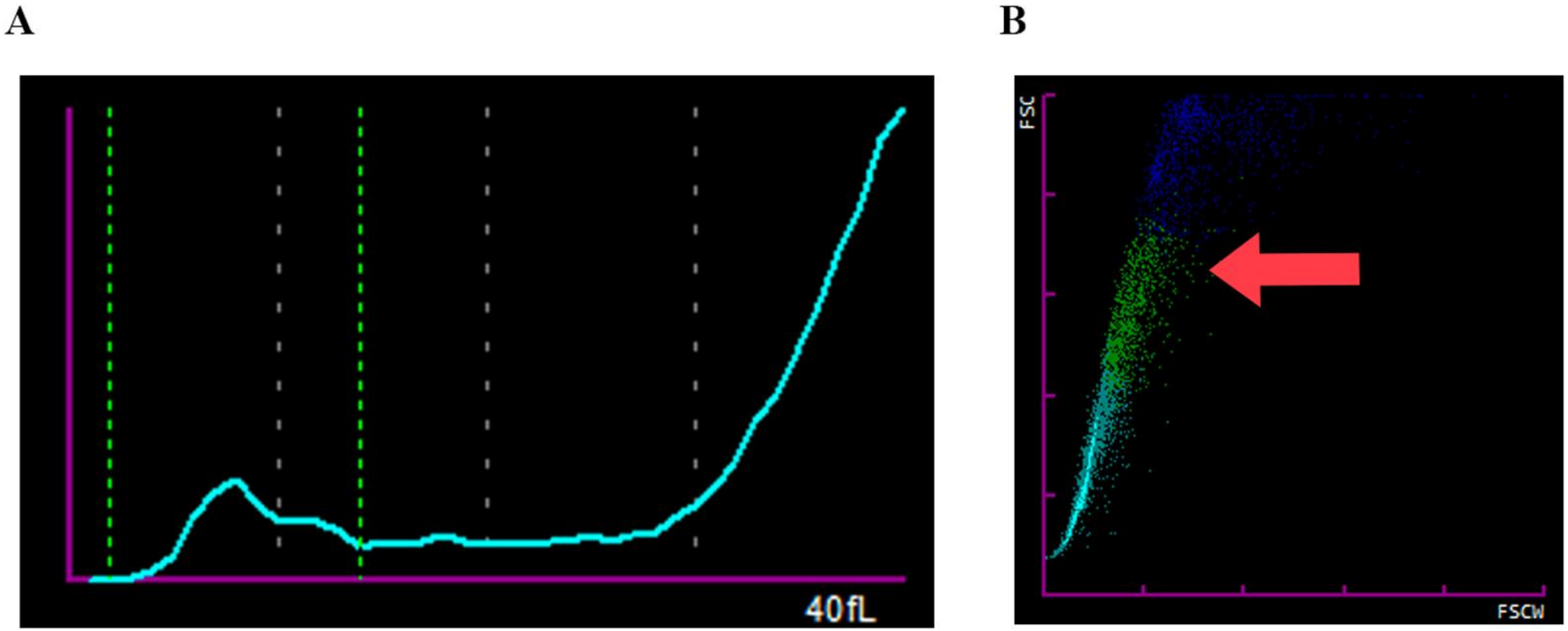

Platelet aggregate interference on the PLT-I histogram and PLT-F scattergram of the Sysmex XN-9000.

(A) PLT-I histogram showing a steep, irregular curve with marked elevation of the tail.

(B) PLT-F scattergram showing increased dispersion of forward scatter width (FSCW), as indicated by the red arrow.
