# Supplementary material for: Multidimensional Tracing of Error Sources and Structured Corrective Strategies for Leukocyte Count and Differential on Automated Hematology Analyzers
Source: Diagnostics (Basel). 2026 Oct 29;16(21):3578. doi: 10.3390/diagnostics16213578 (PMC13761757; doi:10.3390/diagnostics16213578)
Supplement: Supplementary file 1 [file diagnostics-16-03578-s001.zip › Figure S3.pdf]

Figure S3

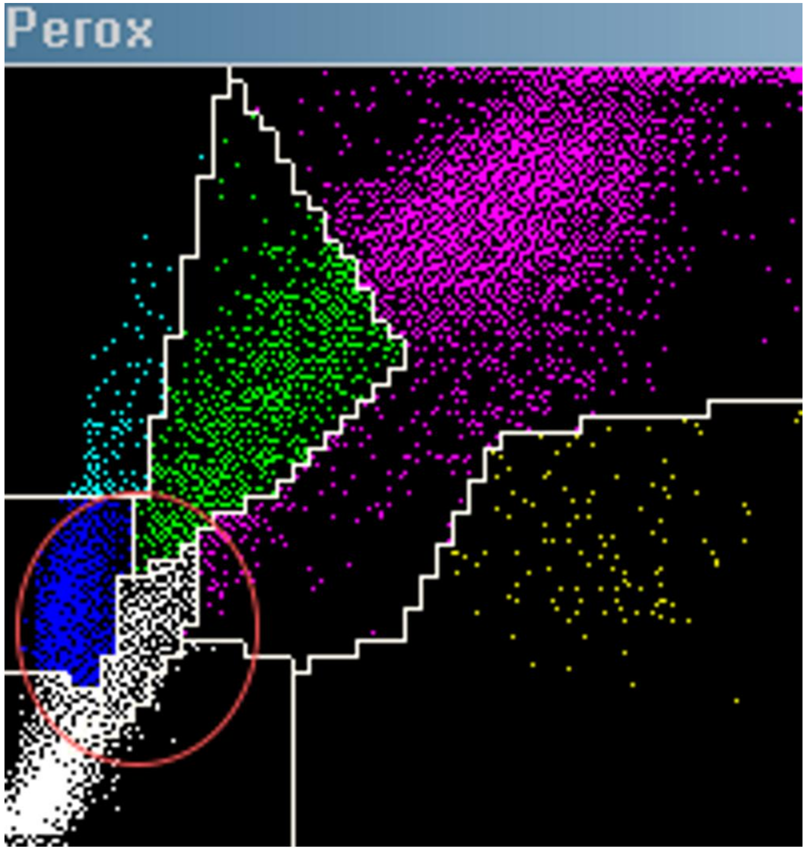

Platelet aggregate interference on the Perox scattergram of the Siemens ADVIA 2120. Numerous abnormal white dot clusters appear within the instrument-defined "noise/platelet clumps" gate and break through the normal cell population boundaries, with some dots connecting to the lymphocyte (dark blue) and monocyte (green) populations; the abnormal area is circled in red.
