# Supplementary material for: Multidimensional Tracing of Error Sources and Structured Corrective Strategies for Leukocyte Count and Differential on Automated Hematology Analyzers
Source: Diagnostics (Basel). 2026 Oct 29;16(21):3578. doi: 10.3390/diagnostics16213578 (PMC13761757; doi:10.3390/diagnostics16213578)
Supplement: Supplementary file 1 [file diagnostics-16-03578-s001.zip › Figure S4.pdf]

Figure S4

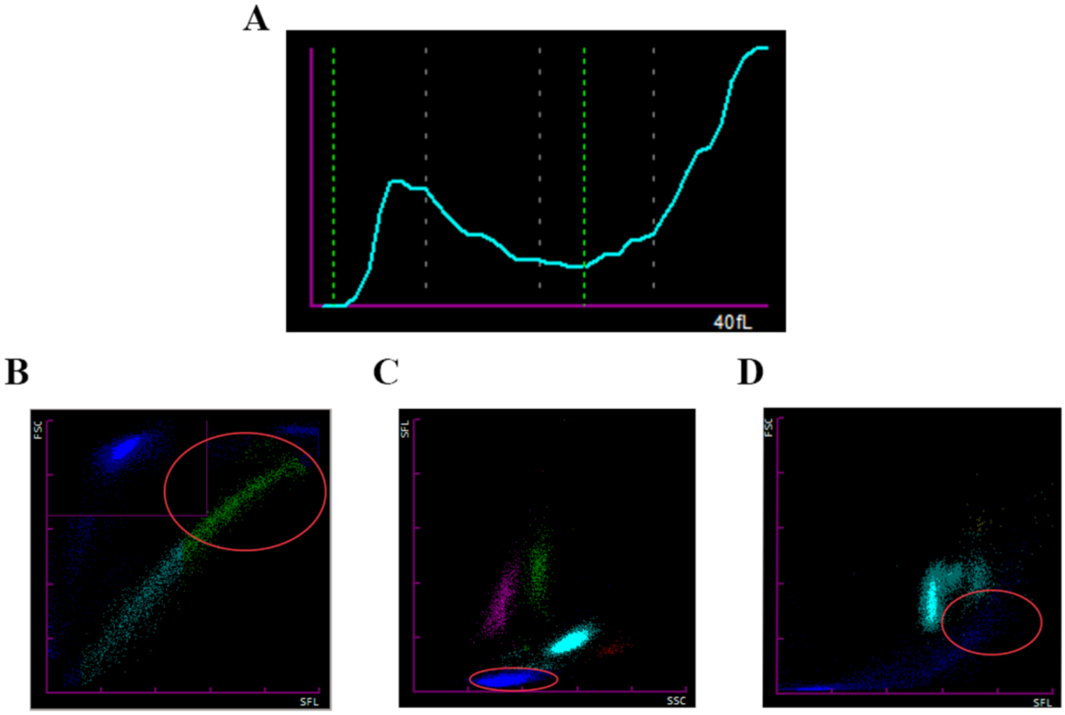

Large platelet interference on the PLT-I histogram, PLT-F, WDF, and WNR scattergrams of the Sysmex XN-9000.

(A) PLT-I histogram showing a low, broad curve with an elevated tail and irregular contour.

(B) PLT-F scattergram showing dots extending toward the region of larger cell volume and stronger fluorescence intensity, abutting the leukocyte population; the abnormal area is circled in red.

(C) WDF scattergram showing numerous dense dark blue dots to the right of the debris area (circled in red).

(D) WNR scattergram showing a marked increase in debris-region dots extending toward the upper right into the leukocyte area; the abnormal area overlapping the leukocyte population is circled in red.
