# Supplementary material for: Multidimensional Tracing of Error Sources and Structured Corrective Strategies for Leukocyte Count and Differential on Automated Hematology Analyzers
Source: Diagnostics (Basel). 2026 Oct 29;16(21):3578. doi: 10.3390/diagnostics16213578 (PMC13761757; doi:10.3390/diagnostics16213578)
Supplement: Supplementary file 1 [file diagnostics-16-03578-s001.zip › Figure S5.pdf]

Figure S5

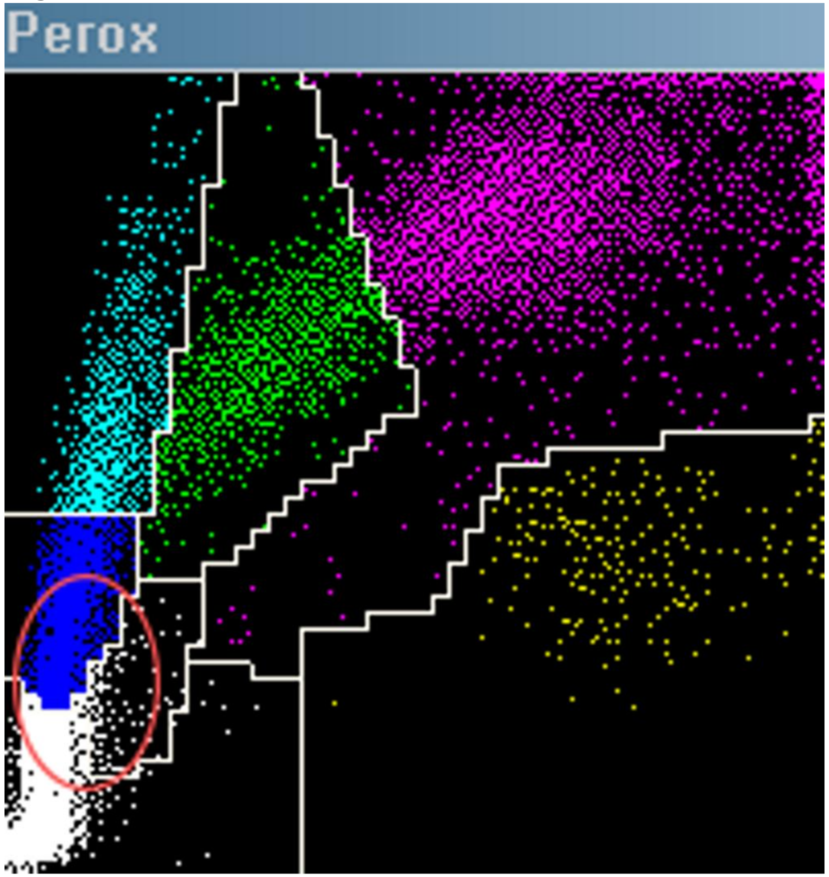

NRBC interference on the Perox scattergram of the Siemens ADVIA 2120. A large population of NRBCs (white dots) connects with the lymphocyte population (dark blue dots) and extends diagonally upward along the lymphocyte cluster, interfering with the lymphocyte differential count; the abnormal area is circled in red.
