# Supplementary material for: Multidimensional Tracing of Error Sources and Structured Corrective Strategies for Leukocyte Count and Differential on Automated Hematology Analyzers
Source: Diagnostics (Basel). 2026 Oct 29;16(21):3578. doi: 10.3390/diagnostics16213578 (PMC13761757; doi:10.3390/diagnostics16213578)
Supplement: Supplementary file 1 [file diagnostics-16-03578-s001.zip › Figure S6.pdf]

Figure S6

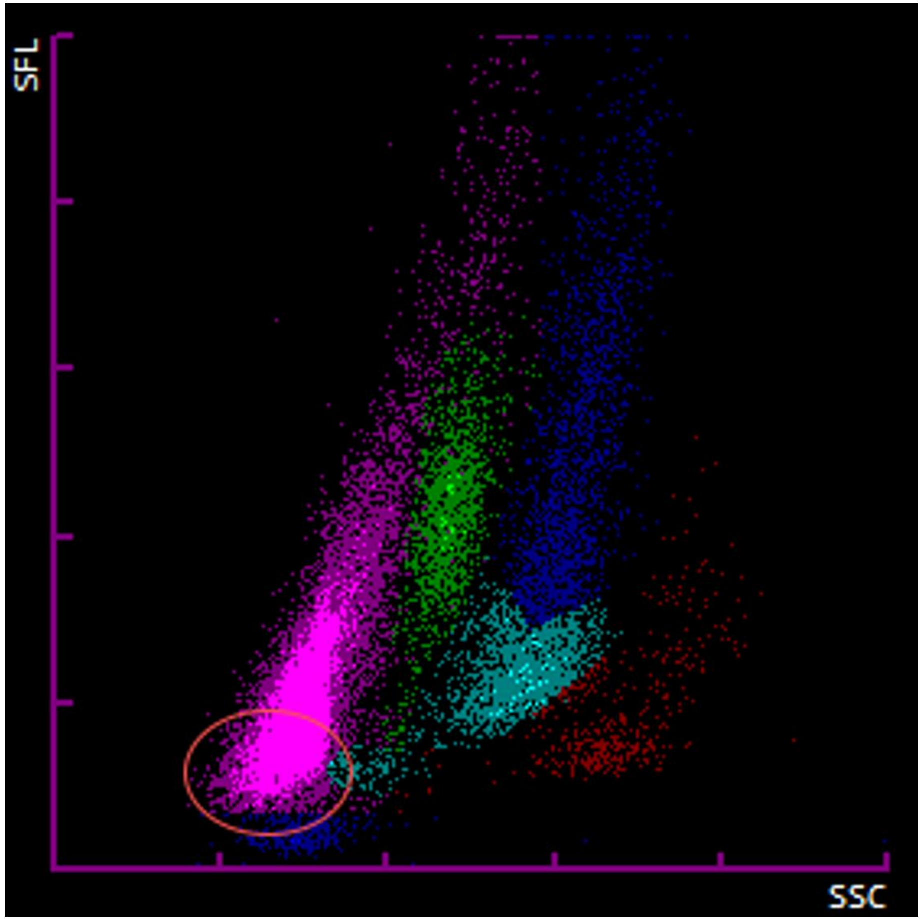

NRBC interference on the WDF scattergram of the Sysmex XN-9000. A population of NRBCs, similar in color to the lymphocytes, connects and merges with the lymphocyte cluster, interfering with lymphocyte differential count; the abnormal area is circled in red.
