# Supplementary material for: Multidimensional Tracing of Error Sources and Structured Corrective Strategies for Leukocyte Count and Differential on Automated Hematology Analyzers
Source: Diagnostics (Basel). 2026 Oct 29;16(21):3578. doi: 10.3390/diagnostics16213578 (PMC13761757; doi:10.3390/diagnostics16213578)
Supplement: Supplementary file 1 [file diagnostics-16-03578-s001.zip › Figure S7.pdf]

Figure S7

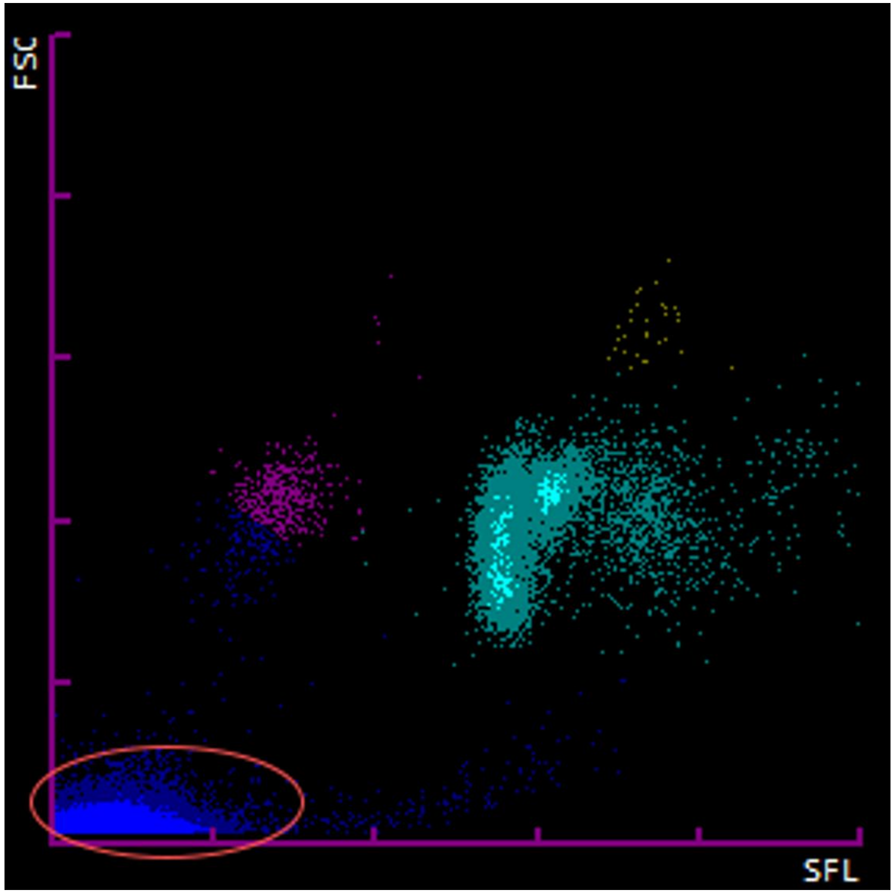

Lysis-resistant RBC interference on the WNR scattergram of the Sysmex XN-9000. A dense abnormal cluster/band of dark blue dots is present adjacent to the X-axis in the lower left corner of the WNR scattergram (circled in red).
