# Supplementary material for: Multidimensional Tracing of Error Sources and Structured Corrective Strategies for Leukocyte Count and Differential on Automated Hematology Analyzers
Source: Diagnostics (Basel). 2026 Oct 29;16(21):3578. doi: 10.3390/diagnostics16213578 (PMC13761757; doi:10.3390/diagnostics16213578)
Supplement: Supplementary file 1 [file diagnostics-16-03578-s001.zip › Figure S8.pdf]

Figure S8

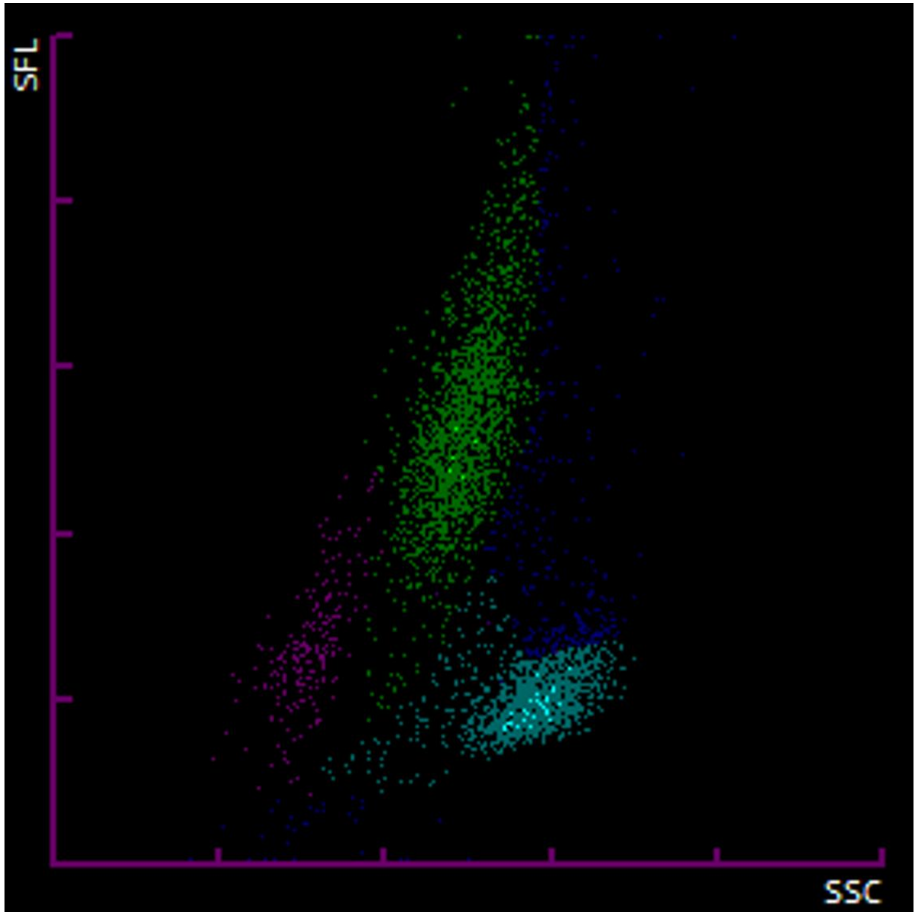

Immature granulocytes misclassified as monocytes on the WDF scattergram of the Sysmex XN-9000. The monocyte population (green) and the immature granulocyte population (dark blue) appear connected and inseparable, leading to misclassification of some immature granulocytes as monocytes.
