# Supplementary material for: Multidimensional Tracing of Error Sources and Structured Corrective Strategies for Leukocyte Count and Differential on Automated Hematology Analyzers
Source: Diagnostics (Basel). 2026 Oct 29;16(21):3578. doi: 10.3390/diagnostics16213578 (PMC13761757; doi:10.3390/diagnostics16213578)
Supplement: Supplementary file 1 [file diagnostics-16-03578-s001.zip › Figure S9.pdf]

Figure S9

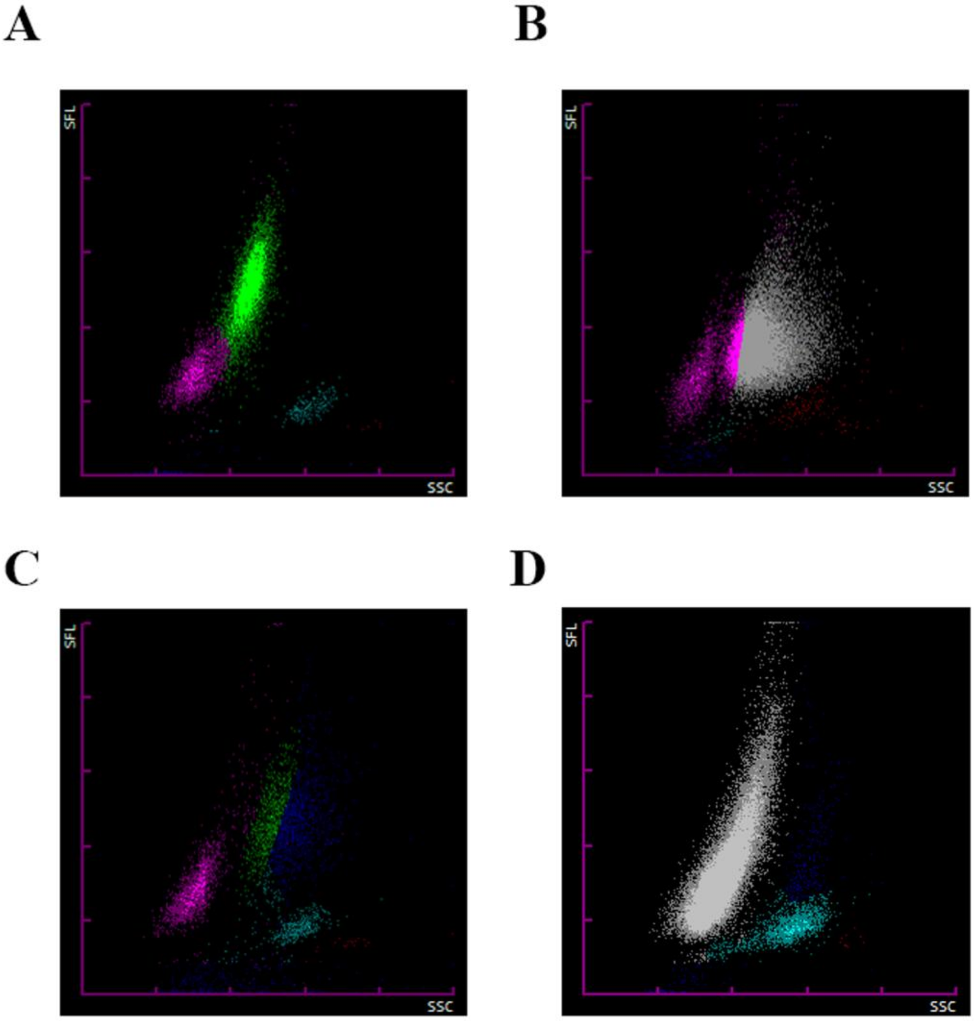

Leukemic cells misclassified as monocytes on the WDF scattergram of the Sysmex XN-9000.

(A) AML-M2: poorly defined boundaries between the lymphocyte and monocyte populations, with an upward shift of the monocyte population.

(B) AML-M3: all populations except lymphocytes merge into a large unclassifiable gray cluster, forming a broad-based, pointed teardrop shape.

(C) AML-M3: the immature granulocyte population shifts leftward and merges with the monocyte population, with a clear tangent line between them.

(D) ALL: increased lymphocyte dots extending into the monocyte region, with the lymphocyte population shifting upward.
