# Supplementary material for: Multidimensional Tracing of Error Sources and Structured Corrective Strategies for Leukocyte Count and Differential on Automated Hematology Analyzers
Source: Diagnostics (Basel). 2026 Oct 29;16(21):3578. doi: 10.3390/diagnostics16213578 (PMC13761757; doi:10.3390/diagnostics16213578)
Supplement: Supplementary file 1 [file diagnostics-16-03578-s001.zip › Supplementary Table S1.pdf]

Supplementary Table S1. Chinese search terms used for CNKI.

Database	Search terms
CNKI	Leukocyte count (白细胞计数); Leukocyte differential count (白细胞分类计数); Leukocyte scattergram (白细胞散点图); Spurious count (假性计数); Pseudoleukocytosis (假性白细胞增多); Pseudo-eosinophilia (假性嗜酸性粒细胞增多); Hyperbilirubinemia (高胆红素血 Hyperglobulinemia (高球蛋白血症); Interference (干扰); Misclassification (纠正)
