# Supplementary material for: Multidimensional Tracing of Error Sources and Structured Corrective Strategies for Leukocyte Count and Differential on Automated Hematology Analyzers
Source: Diagnostics (Basel). 2026 Oct 29;16(21):3578. doi: 10.3390/diagnostics16213578 (PMC13761757; doi:10.3390/diagnostics16213578)
Supplement: Supplementary file 1 [file diagnostics-16-03578-s001.zip › Supplementary Table S2.pdf]

Supplementary Table S2. Detection principles and characteristics of hematology analyzers discussed in this review

Analytical Principle	Platform / Manufacturer	Relevant WBC Channels
**Legacy Generation**		
Impedance (Coulter principle)	Coulter S-Plus IV (Beckman Coulter)	/
	Sysmex KX-21 (Sysmex)	/
	CELL-DYN 1800 (Abbott)	/
Cytochemistry (peroxidase content) and cell volume analysis	Technicon H6000 (Technicon/Bayer)	Peroxidase
Impedance (DC) and radiofrequency (RF)	Sysmex SE-9000 (Sysmex)	DIFF, IMI, WBC/BASO
Fluorescence flow cytometry with semiconductor laser	Sysmex XE-2100 (Sysmex)	DIFF, IMI, WBC/BASO
VCS technology	Beckman Coulter LH750/LH780 (Beckman Coulter)	VCS WBC channel
**Current Generation**		
VCS/VCSn	Beckman Coulter DxH 800/DxH 900 (Beckman Coulter)	VCSn WBC channel
Peroxidase cytochemistry and light scatter	Siemens ADVIA 120 / ADVIA 2120 (Siemens)	Peroxidase, Basophil
SF Cube (3D laser scatter + fluorescence)	Mindray BC-6800 (Mindray)	DIFF, WNB
Sheath-flow impedance combined with semiconductor laser flow cytometry	Sysmex XN-9000/XN-20 (Sysmex)	WNR, WDF, WPC

Note: Hematology analyzers are classified into "Legacy Generation" or "Current Generation" based on their technological era and current clinical prevalence. The symbol "/" indicates no dedicated independent WBC analysis channel.

Abbreviations: WBC, white blood cell; DIFF, WBC differential channel; IMI, immature myeloid information channel; WBC/BASO, WBC/basophil channel; VCS, volume, conductivity, scatter; VCSn, volume, conductivity, scatter, and 5-angle scatter; WNR, white cell nucleated red channel; WDF, white cell differential channel; WPC, white precursor cell channel; WNB, white cell nucleated basophil channel.
